# Dilute Regeneration‐Driven Membrane Capacitive Deionization of Synthetic Seawater using Nanopatterned Membranes and Prussian Blue Analog Electrodes

**DOI:** 10.1002/smll.202510773

**Published:** 2025-11-24

**Authors:** Mahmudul Hasan, Michael Labella, Colton Waters Burke, Christopher G. Arges, Enrique D. Gomez, Christopher A. Gorski

**Affiliations:** ^1^ Department of Chemical Engineering The Pennsylvania State University University Park PA 16802 USA; ^2^ Department of Civil and Environmental Engineering The Pennsylvania State University University Park PA 16802 USA; ^3^ Materials Research Institute The Pennsylvania State University University Park PA 16802 USA; ^4^ Argonne National Laboratory 9700 S. Cass Avenue Lemont IL 60439 USA; ^5^ Department of Materials Science and Engineering The Pennsylvania State University University Park PA 16802 USA

**Keywords:** membrane capacitive deionization (MCDI), nanopatterned ion‐exchange membranes, prussian blue analogs, seawater desalination

## Abstract

Membrane capacitive deionization (MCDI) offers energy‐efficient seawater desalination but is limited at high salinity by membrane resistance and incomplete electrode regeneration. Nanopatterned ion‐exchange membranes, dilute regeneration protocols, and Prussian blue analog (PBA)‐functionalized electrodes are combined in a flow‐by‐MCDI cell. Nanopatterned ion‐exchange membranes (hexagonal, octagonal, double‐ring, rectangular) enhance interfacial ion transport, with hexagonal geometry delivering ≈12.5% greater surface area and the best performance. PBA‐functionalized electrodes increase salt adsorption and charge‐transfer kinetic rates. The integrated system lowers the area‐specific resistance by 45 Ω cm^2^, resulting in a 500 mV reduction in the cell voltage for a current density of 2 mA cm^−2^ for a 35 000 ppm NaCl feed. This improves the energy‐normalized salt adsorption six fold (64–382 mmol J^−1^). Low salinity (2000 ppm) and mixed‐salt regeneration sustains a ≈39% water recovery and stable performance for at least seven cycles. Overall, combining nanopatterned membranes, which promote confinement‐enhanced ion mobility, and PBA electrodes, which enhance salt adsorption, improved the energy efficiency of MCDI.

## Graphical abstract

This work presents a novel strategy for synthetic seawater desalination by combining dilute regeneration protocols, nanopatterned ion‐exchange membranes, and Prussian blue analog‐coated electrodes. This integrated approach reduces ohmic resistance and enhances mass transfer by inducing secondary flows along the membrane surface, leading to improved salt removal efficiency and system stability.

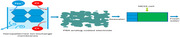

## 1 Introduction

The rising demand for freshwater, fueled by rapid population growth, climate change, and industrial development, has turned water scarcity into a pressing global issue. Although ≈97% of Earth's water is in the oceans, its high salinity makes it unsuitable for human consumption.^[^ ^1^, ^2^, ^3^ ^]^ This challenge has driven advancements in desalination technologies, especially for coastal regions where seawater is abundant.^[^ ^4^, ^5^, ^6^, ^7^ ^]^

Membrane capacitive deionization (MCDI) has emerged as a promising electrochemical desalination technique for brackish water treatment. Relative to conventional desalination methods (e.g., reverse osmosis and thermal distillation), MCDI is unique in that it is modular, allowing for simple scalability and the ability to operate at a large concentration gradient in a single stage.^[^ ^8^, ^9^, ^10^, ^11^ ^]^ In MCDI, a flow‐by configuration is often employed in which ion‐selective membranes cover porous carbon electrodes.^[^ ^12^, ^13^, ^14^ ^]^ When an electric field is applied, dissolved ions are driven through ion‐selective membranes and captured by charged porous carbon electrodes.^[^ ^15^, ^16^, ^17^ ^]^ More specifically, in MCDI, the cation‐exchange membrane (CEM) and anion‐exchange membrane (AEM) operate through electrostatic (Donnan) exclusion and ion‐exchange mechanisms inherent to their fixed charged groups. The CEM, containing negatively charged sulfonic acid groups, selectively allows cations (e.g., Na⁺, Mg^2^⁺) to pass while repelling anions. Conversely, the AEM, functionalized with positively charged quaternary ammonium groups, facilitates anion (e.g., Cl^−^, SO_4_^2^^−^) transport while excluding cations. This charge‐based selectivity governs ion transport across the membranes during the deionization in the MCDI process.

**Figure 1:**
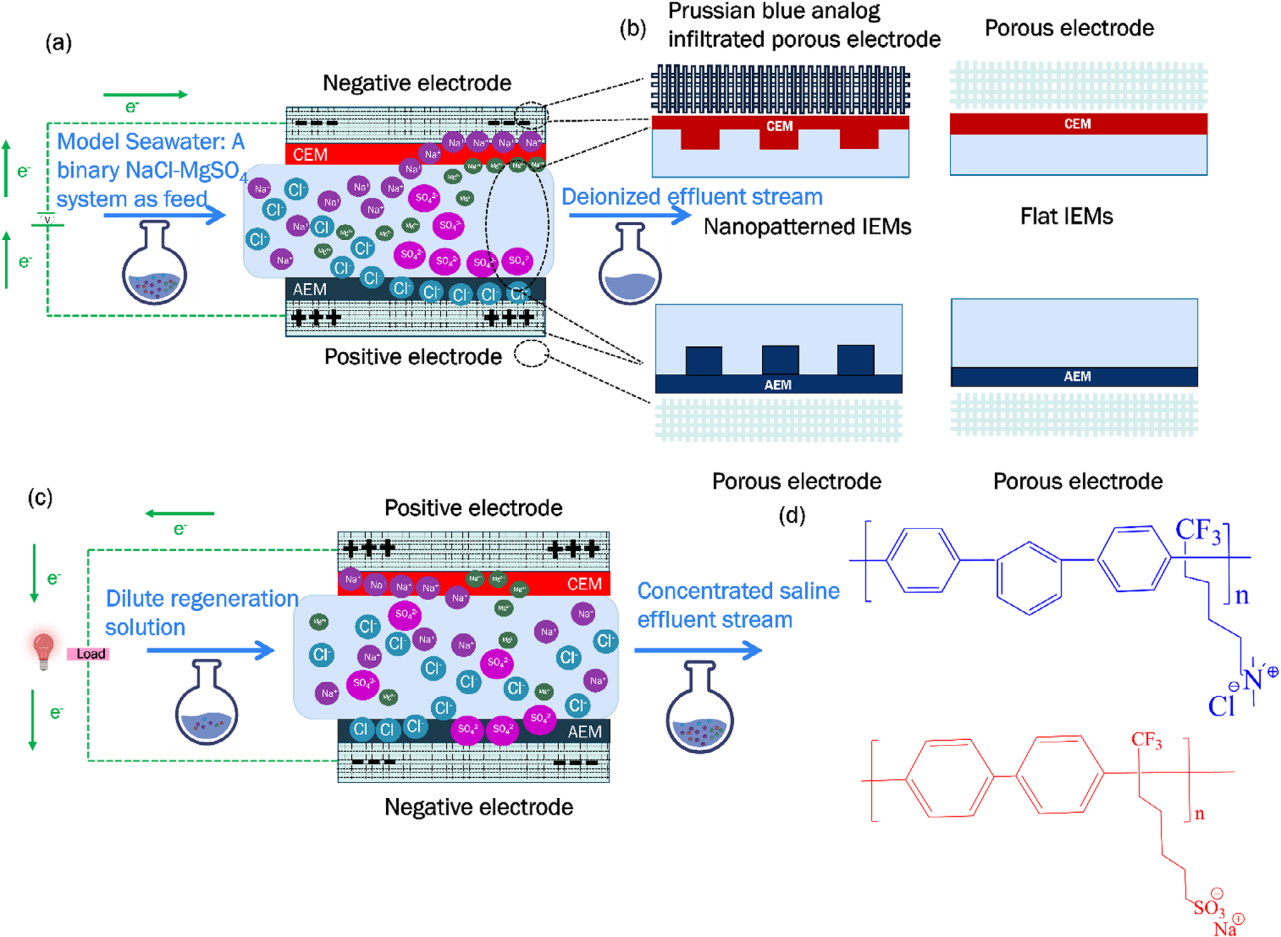
a) MCDI process during deionization (i.e., charge step) for model seawater; b) MCDI with nano‐patterned IEMs and Prussian blue analog‐infiltrated porous electrodes to decrease ohmic resistances within the cell; c) MCDI during electrode regeneration (i.e., discharge step) with a dilute regeneration solution; d) Chemical structures of the AEM (top, blue) and the CEM (bottom, red) used in this study.

During the regeneration in MCDI, the ions are released from the electrodes and migrate across the said membranes. The entropy gain during the regeneration step manifests a free energy release that can be recovered and applied in a subsequent deionization cycle.^[^ ^18^, ^19^ ^]^ **Figure** **1**a illustrates the architecture of the flow and deionization process within an MCDI system.

However, for MCDI to be a competitive option for seawater desalination, several performance bottlenecks must be addressed. This work addresses two: 1) the high resistance of ion‐exchange membranes, which increases the energy demand, and 2) the limited salt storage capacity of conventional carbon cloth electrodes, which results in energetic inefficiencies during frequent switching between concentrate and dilute stream flows through the cell.^[^ ^20^, ^21^ ^]^ Complete electrode regeneration—defined as total ion removal from the electrodes—is necessary for seawater desalination to remove a sufficient amount of the salt, but this mode of operation increases energy demands. In this work, we evaluated the effectiveness of three approaches to enhance MCDI performance in seawater desalination: 1) developing improved ion‐exchange membranes, 2) achieving greater desalination by increasing the salt storage capacities of electrodes, and 3) decreasing the salinity of the rinse solution below that of seawater. Figure 1c depicts electrode regeneration in the MCDI system, driven by a dilute regeneration solution.

In this work, we considered other ions, such as magnesium (Mg^2^⁺) and sulfate (SO_4_^2^^−^), which are found in seawater but at lower concentrations compared to the abundant ions sodium (Na⁺) and chloride (Cl^−^). Mg^2^⁺ and SO_4_^2^^−^ contribute significantly to the overall ionic strength and salinity of seawater.^[^ ^22^, ^23^ ^]^ Hence, examining these ions in the saline solution is important to understanding MCDI performance and performance limitations with model seawater solutions.

Driven by the need to reduce membrane resistance in MCDI, recent research has turned toward the development of high‐performance ion‐exchange membranes.^[^ ^24^, ^25^, ^26^, ^27^, ^28^ ^]^ These membranes play a pivotal role in electrochemical desalination by enabling the selective transport of cations or anions while efficiently blocking counter‐ions and impurities. Their performance hinges on a delicate balance of key properties, including ionic conductivity, water permeability, electro‐osmotic drag, mechanical integrity, and resistance to fouling. To meet these competing requirements, a new generation of advanced membranes has emerged. Materials incorporating graphene oxide, carbon nanotubes, or bioinspired structures offer enhanced durability, ion selectivity, and salt rejection.^[^ ^29^, ^30^, ^31^, ^32^, ^33^ ^]^ Furthermore, hybrid membranes that combine polymeric and inorganic materials enhance both the performance and sustainability of electrochemical desalination technologies.^[^ ^34^, ^35^, ^36^, ^37^ ^]^

While advances in membrane materials have significantly improved durability and selectivity, optimizing membrane performance in MCDI systems remains challenging due to inherent trade‐offs among key properties such as thickness, ionic conductivity, and selectivity. For instance, reducing membrane thickness can lower ionic resistance but also tends to decrease permselectivity, diminishing overall separation performance. To overcome these trade‐offs, we introduce surface patterning as a physical design strategy that increases the membrane–water interfacial area without reducing membrane thickness or compromising its mechanical strength. Furthermore, nano‐ and micro‐scale surface patterning has demonstrated the ability to enhance mass transport and reduce concentration polarization by generating local secondary flows and thinning boundary layers. Prior studies have shown that patterned membranes can modulate local hydrodynamics, reduce osmotic pressure buildup, and enhance desalination performance.^[^ ^38^, ^39^, ^40^ ^]^

Creating surface‐patterns on membranes promotes water permeability and creates preferential pathways for water transport while selectively repelling salt ions and foulants with high precision.^[^ ^41^, ^42^ ^]^ Advances in fabrication techniques, such as nanoimprint lithography and direct pattern transfer via transfer printing, have enabled the production of membranes with tailored surface topographies.^[^ ^43^, ^44^ ^]^ Patterning membranes enhances their longevity and performance in electrochemical energy storage and conversion devices that rely on ion exchange membranes.^[^ ^45^ ^]^ The use of surface‐patterned membranes in proton exchange membrane fuel cells enhanced energy output by promoting efficient proton transport and minimizing fuel crossover.^[^ ^46^, ^47^, ^48^ ^]^ In our previous work, we integrated micropatterned IEMs with 20 µm cylindrical wells into a flow‐by MCDI cell, resulting in a reduction of 700 mV in operating voltage during brackish water desalination.^[^ ^49^ ^]^ In the present work, we examined the benefits of surface‐patterned membranes for seawater desalination and examined how the use of nano‐scale patterning, as opposed to micron‐scale patterning, improved overall MCDI performance. The integrated design of MCDI with flat and nanopatterned anion and cation exchange membranes (AEMs and CEMs), along with electrodes infiltrated with Prussian blue analogs, is depicted in Figure 1c, and the chemical structure of the AEM and CEM is shown in Figure 1d.

Conventional porous carbon electrodes used in MCDI systems suffer from limited salt storage capacity and require near‐complete regeneration for effective operation, significantly increasing energy consumption. To overcome this, Prussian blue analog (PBA) electrodes offer a promising alternative due to their unique crystalline framework, high density of redox‐active sites, and the ability to selectively intercalate cations such as Na⁺ and Mg^2^⁺.^[^ ^50^, ^51^, ^52^, ^53^, ^54^, ^55^ ^]^ These properties allow PBAs to achieve higher ion storage capacities with improved reversibility, making them particularly effective in high‐salinity waters, such as seawater.^[^ ^56^, ^57^, ^58^ ^]^ Recent literature has focused on overcoming primary limitations, namely low capacity and cyclic instability, of PBA electrode materials. For instance, enhanced crystallization strategies have successfully minimized inherent structural vacancies, leading to exceptionally high salt adsorption capacities (SAC) of up to 101.4 mg g^−1^.^[^ ^59^ ^]^ Simultaneously, entropy engineering has been employed to constrain undesirable multistage phase transitions, dramatically improving cycling performance and enabling ultralong‐life stability.^[^ ^60^ ^]^ Beyond capacity and stability enhancements, PBAs are versatile: they are utilized in advanced architectures, such as freestanding MXene/PBA films for hybrid CDI (HCDI) with favorable kinetics and for the selective removal of specific ions, including ammonium (NH_4_^+^) from wastewater.^[^ ^61^, ^62^ ^]^ This established utility of PBAs for high‐performance ion capture justifies their selection as the active material in our study to maximize the efficiency of the nanopatterned membrane system.

To increase ion desorption efficiency during electrode regeneration, we examined the effect of the salinity of the rinse solutions. Using a lower‐salinity rinse promotes stronger concentration gradients across the electrode–membrane‐fluid interface, thereby accelerating ion release kinetics and facilitating more efficient electrode recovery. To the best of our knowledge, this paper is the first to implement a salinity‐optimized rinsing protocol for enhancing regeneration efficiency in MCDI.

In this study, we hypothesized that nanoscale surface patterning could further reduce energy losses in MCDI systems treating seawater‐level salinities due to the availability of more active sites for ion transport and enhanced size‐exclusion effects from the nanopore geometry. To investigate this, we explored three promising strategies to enhance the efficiency of MCDI seawater desalination: 1) employing nanopatterned ion‐exchange membranes with varied geometries to minimize membrane resistance (i.e., hexagonal, double‐ring, octagonal, and rectangular patterns ranging from 100 to 300 nm), 2) substituting conventional porous carbon electrodes with PBA‐based electrodes with high salt storage capacities,^[^ ^63^, ^64^ ^]^ and 3) varying the salinity of the rinse solutions.

We introduce an operational strategy that uses dilute NaCl regeneration solutions (0–5 g L^−1^) and dilute mixtures of NaCl and MgSO4 solutions to treat 35 g L^−1^ NaCl and a mixture of 30 g L^−1^ NaCl and 5 g/L^−1^ MgSO_4_, respectively. A recent study employed a similar range of NaCl concentrations, aiming to concentrate saline water as part of a zero liquid discharge approach.^[^ ^65^ ^]^ In contrast, our strategy enabled efficient and complete restoration of electrode functionality, highlighting its potential for sustained electrochemical performance for desalination.

## 2 Results and Discussion

### 2.1 Nanopatterned Membrane Fabrication and Characterization

**Figure 2:**
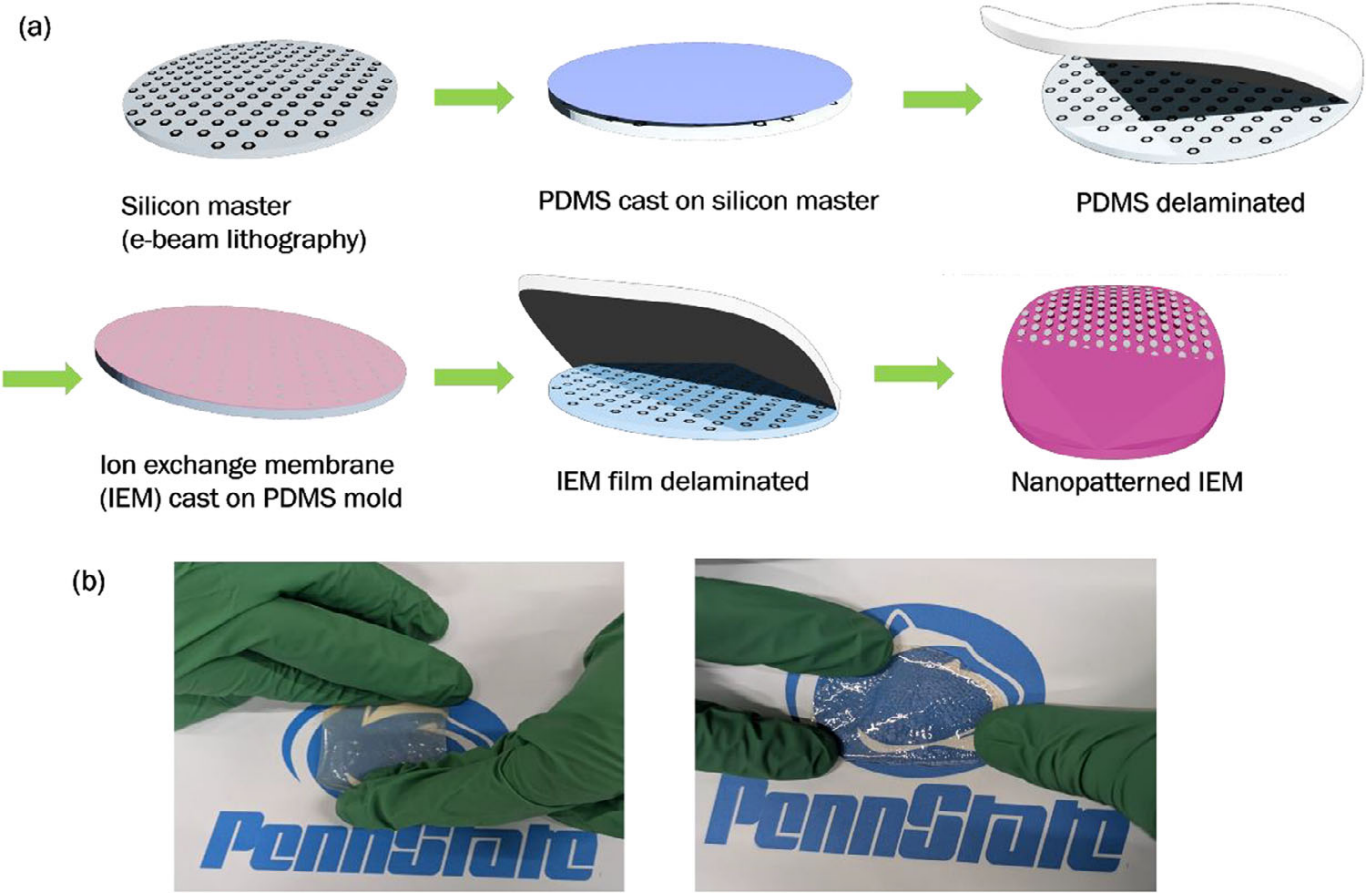
a) Process flow for fabricating nanopatterned AEMs and CEMs with systematically varying interfacial areas in the junction region. b) Images of the 100 nm × 75 nm concentric hexagonal patterned poly (phenylene alkylene) AEM (left) and CEM (right).

Nanopatterned ion exchange membranes (IEMs) were fabricated by first defining hexagonal, octagonal, rectangular, and double‐ring patterns on a silicon master with an e‐beam resist using electron beam lithography, followed by transferring these designs onto a poly(dimethylsiloxane) (PDMS) submaster mold.^[^ ^66^, ^67^, ^68^ ^]^ Subsequently, poly(phenylene alkylene) ionomer solutions were drop‐cast onto the nanopatterned PDMS submaster mold, yielding nanopatterned AEMs and CEMs. The PDMS submaster mold supports multiple replications of nanopatterned AEMs and CEMs. Four different types of surface‐patterned IEMs were fabricated: 100 nm × 75 nm concentric hexagonal wells, 100 nm × 75 nm concentric octagonal wells, 100 nm × 75 nm concentric rectangular wells, and 100 nm diameter double ring wells. The fabrication steps and the resulting nanopatterned membranes are shown in **Figure** **2** (see Sections S1 and S2, Supporting Information).

**Figure 3:**
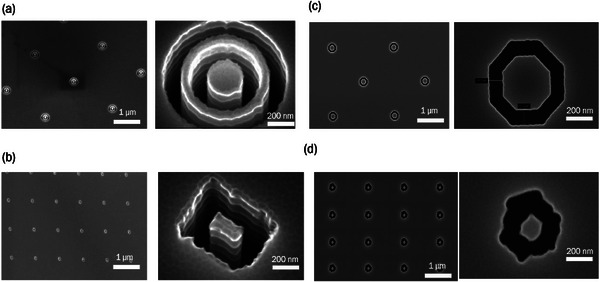
Top‐down scanning electron micrographs of the a) double rings, b) concentric rectangular, c) concentric octagonal, and d) concentric hexagonal nanopatterned photoresist on the silicon master substrate.

Each nanopattern feature defines inner geometries (i.e., 60 nm × 40 nm hexagonal inner wells, 60 nm × 40 nm octagonal inner wells, 60 nm × 40 nm rectangular inner wells, etc.). Although the surface geometries varied, the periodic spacing and lateral feature dimensions were deliberately kept constant to isolate changes in MCDI performance to geometric effects alone. To clarify, the nanometer‐scale dimensions reported here (e.g., 100 nm × 75 nm) refer to the lateral dimensions of the lithographically defined surface nanopatterns, not to the intrinsic hydrated pore size of the membrane. The hydrated pore size in the poly(phenylene alkylene)‐based ion‐exchange membrane is in the sub‐nanometer to few‐nanometer range (0.3–1.1 nm) as reported in the literature.^[^ ^69^ ^]^ This nanofabrication approach was validated by confirming the successful transfer of nanopatterns from the silicon master to the IEMs using scanning electron microscopy (SEM, **Figure** **3**) and atomic force microscopy (AFM, Figure S3, Supporting Information). Beyond revealing lateral feature dimensions, the topographical AFM maps were also used to quantify the amount of increased surface area. The nanopatterns increase the membrane surface area by 12.5% with concentric hexagons, followed by 5.0% for double rings, 3.5% for concentric octagons, and 3.0% for concentric rectangles (Figure S3, Supporting Information). Key IEM properties such as ion exchange capacity (IEC), chemical structure confirmed by NMR, water uptake, and ionic conductivity, were collected and presented in Figures S4 and S5, and Table S1 (Supporting Information).^[^ ^70^, ^71^ ^]^ The data in Table S1 (Supporting Information) show that biphenyl n‐alkyl sulfonic acid CEMs consistently have higher IEC and conductivity compared to meta‐terphenyl n‐alkyl quaternary ammonium (m‐TPN1) AEMs across all geometries. Geometric patterning—such as concentric hexagonal, octagonal, double ring, and rectangular designs—generally leads to increased water uptake (WU) and, in most cases, enhanced conductivity, especially for CEMs. For instance, the concentric hexagonal BPSA CEM has higher conductivity (31.3 mS cm^−1^) than its unpatterned counterpart (29.8 mS cm^−1^), along with slightly improved WU and IEC. The variation in ion permeability among the differently patterned membranes arises from differences in surface topography and effective interfacial area. As shown by AFM in Figure S3 (Supporting Information), each nanopattern geometry (hexagonal, octagonal, rectangular, and double ring) introduces distinct nanoscale surface curvatures and feature densities. These alter the local electric field distribution, ion concentration polarization, and hydration layer structure at the membrane–solution interface. These results suggest that increasing membrane surface area through patterning can improve transport properties without significantly altering IEC or swelling ratio (SR).

### 2.2 Tuning Concentration of Rinse Solution when Desalinating Synthetic Seawater

**Figure 4:**
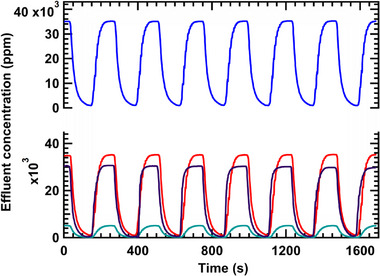
Effluent concentration profile in MCDI for: a) 35 000 ppm NaCl (blue), b) a homogeneous mixture of 30 000 ppm NaCl and 5000 ppm MgSO_4_ (red), 5000 ppm MgSO_4_ (green) and 30 000 ppm NaCl (purple) separately using flat poly(phenylene alkylene) IEMs and porous carbon cloth electrodes at a current density of 2 mA cm^−2^. The regeneration solution was DI water (0 ppm NaCl). Both the charge (deionization) and discharge (regeneration) steps were 120 s each. Notably, the deionization profile of the 30 000 ppm NaCl and 5000 ppm MgSO_4_ mixture (red) closely resembles the sum of individual profiles for NaCl (purple) and MgSO_4_ (green).

Following the successful fabrication of poly(phenylene alkylene) IEMs with well‐defined surface patterns, we initially performed benchmarking experiments using flat IEMs for the deionization of 35 000 ppm salt solutions. The benchmarking was necessary due to the limited data available for MCDI performance at such high salinity feeds and with relevant Mg^2+^ concentrations. The deionization results for a 35 000 ppm NaCl solution and a mixed solution containing 30 000 ppm NaCl and 5000 ppm MgSO_4_, using deionized water as the regeneration medium, are presented in **Figure** **4**. This regeneration strategy is called dilute regeneration, as the ion adsorption capacity of electrodes is restored by flushing the MCDI/CDI cell with a low‐concentration salt solution (or deionized water) during the desorption (regeneration) phase. The concentration drops from 35 000 to 1000 ppm using the dilute regeneration process, outperforming conventional membrane capacitive deionization, in which regeneration was done with 35 000 ppm NaCl.^[^ ^72^, ^73^, ^74^, ^75^ ^]^ This improvement is attributed to the higher ion release to dilute solutions at the electrode surface driven by a favorable concentration gradient. Also, without regeneration, the effluent concentration drop for such a high saline feed is not significant and stable over the cycles at less than 1 V. Overpotential membrane capacitive deionization (>2 V) might improve the deionization a little bit without regeneration, but it will also be harmful for the device in the long run as high potentials spur corrosion and isn't even comparable to the scenario with regeneration. The difference between effluent concentration with and without regeneration is shown in SI 14 of the supplementary information.

**Figure 5:**
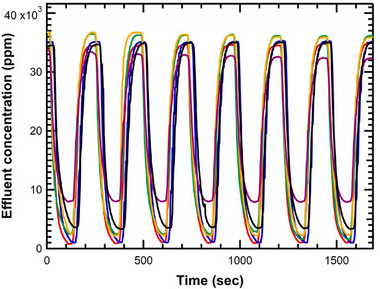
Effluent concentration profile in MCDI for 35 000 ppm NaCl while flushing with a) deionized water (blue), b) 250 ppm NaCl (red), c) 500 ppm NaCl (green), d) 1000 ppm NaCl (yellow), e) 2000 ppm NaCl (black) and f) 5000 ppm NaCl (purple) using flat poly(phenylene alkylene) IEMs and porous carbon cloth electrodes at a current density of 2 mA cm^−2^. Both the charge (deionization) and discharge (regeneration) steps were 120 s each.

We hypothesized that decreasing the salinity of the regeneration (flushing) solution would improve electrode regeneration by enhancing ion desorption due to a stronger concentration gradient between the electrode surface and the rinse solution. However, using lower‐salinity water—particularly deionized (DI) water—for regeneration poses a tradeoff, as it reduces overall water recovery due to the use of larger volumes of low‐salinity water. To investigate this tradeoff, we tested flushing solutions with varying NaCl concentrations, from 0 ppm (DI water) to 5000 ppm. The salt concentration of the flushing water influenced the salt removal efficiency and water recovery for deionization (**Figure** **5**). As the flushing concentration increased from DI water (blue) to 5000 ppm NaCl (purple), the regeneration became progressively less effective, resulting in higher baseline effluent concentrations and reduced deionization efficiency. Between 0 to 2000 ppm NaCl, there was little change in deionization performance; therefore, 2000 ppm was selected as the regeneration concentration for subsequent experiments. These trends highlight the importance of regeneration solution composition in sustained MCDI performance over multiple cycles, particularly under high‐salinity conditions.

To better replicate the composition of seawater, 5000 ppm MgSO_4_ was added to 30 000 ppm NaCl, maintaining a constant total ion concentration of 35 000 ppm. During deionization, a regeneration solution containing 1700 ppm NaCl and 300 ppm MgSO_4_ was used, preserving a total ion concentration of 2000 ppm. Figure S9 (Supporting Information) depicts the separate deionization of MgSO_4_ and NaCl, demonstrating the cell's effectiveness in selectively capturing Mg^2^⁺ and SO_4_^2^^−^ ions. All experiments shown in Figures 4 and 5, and Figure S9 (Supporting Information) were conducted using flat poly(phenylene alkylene) and porous carbon cloth electrodes within the MCDI cell at a current density of 2 mA cm^−2^. Salt removal efficiency (SRE), water recovery (WR), and average salt adsorption rate (ASAR) are summarized in Tables S3–S5 (Supporting Information), respectively, based on data from Figures 4 and 5, and Figure S9 (Supporting Information). The results indicate that SRE remained consistently high (>96%) across most feed types, with the highest SRE of 98.73% achieved for 30 000 ppm NaCl (Table S3, Supporting Information), and the highest ASAR of 56.36 mmol·m^−^^2^·s^−1^ observed for a mixed salt feed flushed with 1700 ppm NaCl and 300 ppm MgSO_4_ (Table S5, Supporting Information). Water recovery ranged from 28.72% to 40.42%, with the highest recovery occurring under mixed feed flushing conditions (Table S5, Supporting Information). The formulas used to calculate these performance metrics are provided in Section S8 (Supporting Information).

As shown in Table S3 (Supporting Information), increasing the concentration of the regeneration solution leads to a noticeable decline in salt removal efficiency, with the lowest performance observed when flushing with 5000 ppm NaCl. This trend can be attributed to the greater availability of ions at higher concentrations, which promotes ion accumulation near the electrode surface and increases the thickness of the electrical double layer, thereby reducing ion removal efficiency.^[^ ^76^ ^]^

**Figure 6:**
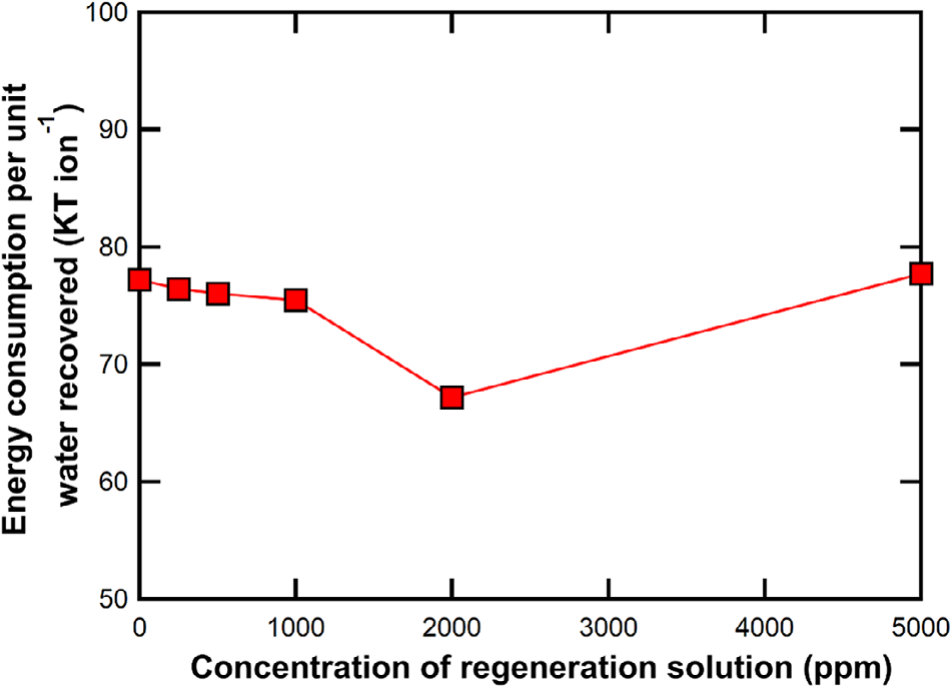
Energy consumption per unit water recovered for different concentrations of regeneration solution.

The water recovery significantly improves, reaching up to ≈39%, when dilute regeneration solutions (0–250 ppm NaCl) are used (Tables S2 and S3, Supporting Information). Also, energy consumed per unit water recovered is lowest (67.2 kT ion^−1^) for 2000 ppm regeneration solution, as can be seen in **Figure** **6**. It increases again when the regeneration solution concentration is increased to 5000 ppm. Thus, to balance both salt removal efficiency and water recovery, a regeneration solution concentration of 2000 ppm NaCl was selected for all subsequent experiments.

### 2.3 Performance of Nanopatterned IEMs and Prussian Blue Analog Infiltrated Electrodes

**Figure 7:**
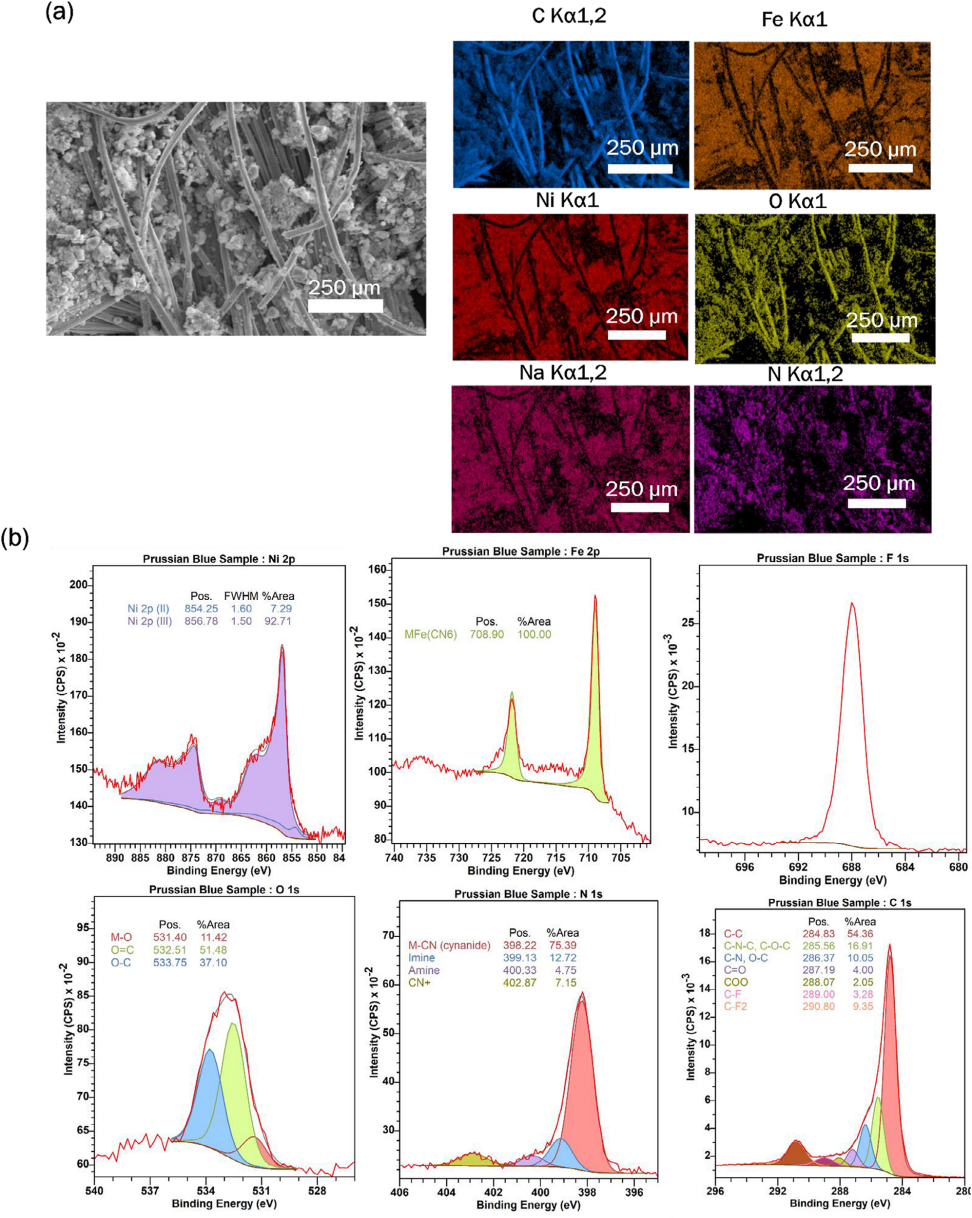
a) Electron micrograph (left) and Energy Dispersive X‐ray Spectroscopy (EDS) elemental maps (right) for iron, nickel, sodium, and nitrogen resembling Prussian blue analog coating on porous carbon cloth electrodes. b) Surface composition analysis of different elements such as nickel, iron, nitrogen, and oxygen by X‐ray photoelectron spectroscopy (XPS).

Alongside nanopatterned membranes, electrodes coated with Prussian blue analogs were developed to further reduce the energy demands for seawater desalination. Prussian blue and its analogs (PBAs) are redox‐active materials known for their open framework structures, which enable efficient ion intercalation and deintercalation. Studies have demonstrated that electrodes modified with PBA exhibit higher salt adsorption capacities and improved charge redistribution relative to carbon‐based electrodes.^[^ ^77^, ^78^, ^79^, ^80^ ^]^ A nickel PBA (NaNi[Fe(CN)_6_]·nH_2_O) was mixed with PVDF and conductive carbon in an 8:1:1 ratio in N‐methyl‐2‐pyrrolidone and coated onto activated carbon cloth electrodes. Then, the infiltrated electrodes were dried in an oven at 60 °C for 2 h to remove the solvent. Elemental mapping using energy‐dispersive X‐ray spectroscopy (EDS) in a scanning electron microscope (SEM) confirmed the presence and uniform distribution of the PBA within the porous electrode matrix (**Figure** **7**). Elemental mapping confirmed the presence of sodium, nickel, iron, and nitrogen on the porous fiber network, consistent with the nickel PBA being incorporated in the coating. XPS confirms the successful formation of nickel‐iron Prussian Blue Analog (PBA) on the carbon cloth, with Ni (2.7%) and Fe (2.4%) compositions consistent with the expected stoichiometry. The presence of nitrogen (7.8%) reflects cyanide ligands within the framework, while fluorine (12.8%) originates from the PVDF binder. Also, trace sodium (0.4%) reflects residual ions from the synthesis of PBA analog powder. Overall, the surface composition aligns well with the characteristic profile of PBA‐coated electrodes.

**Figure 8:**
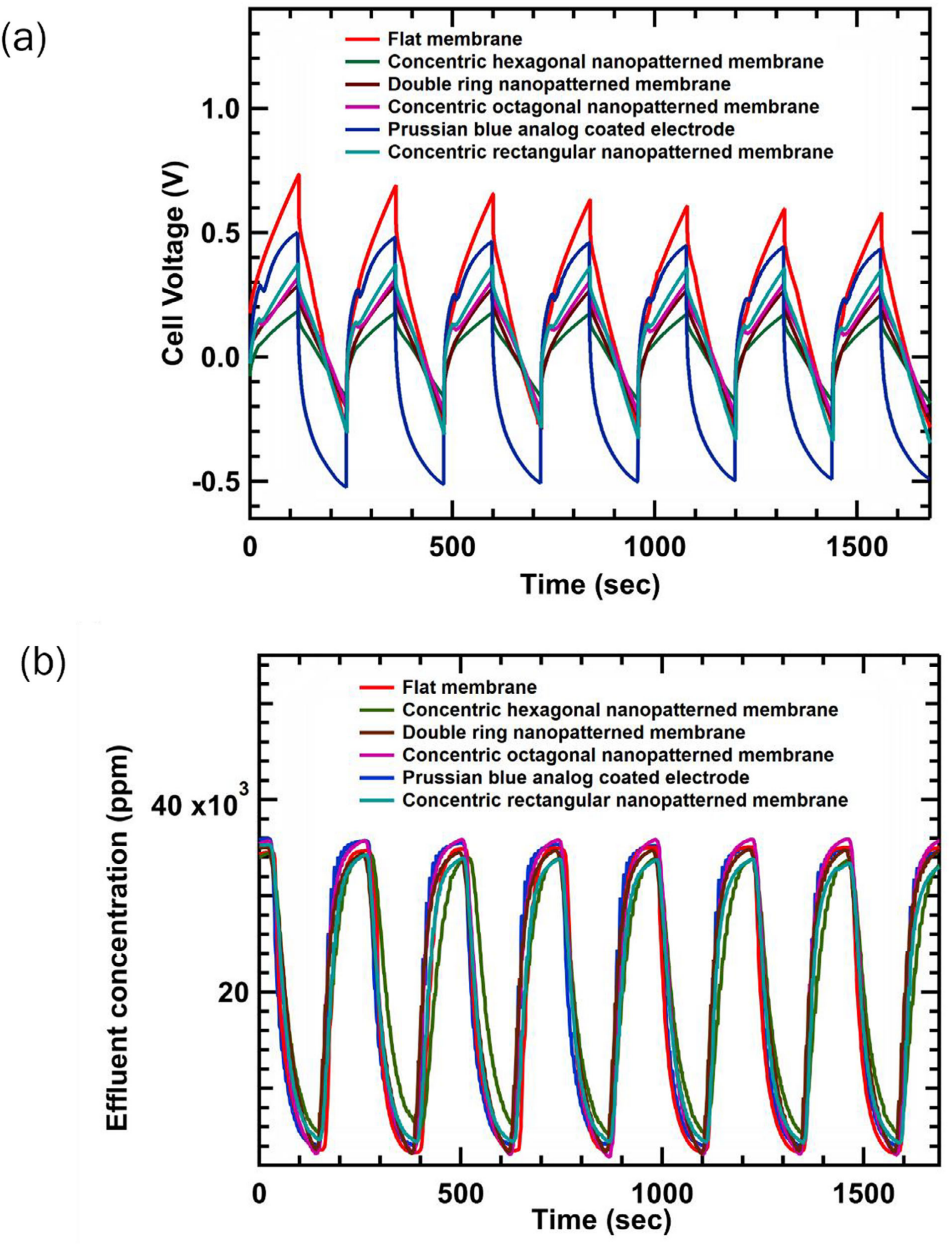
a) Cell voltage vs time and b) effluent concentration profiles vs time for charge–discharge cycles in MCDI at 2 mA cm^−^^2^ for a 35 000 ppm NaCl feed using flat and nanopatterned IEMs. Both the charge (deionization) and discharge (regeneration) steps were 120 s each. The cell geometric projected area was 25 cm^2^. Seven cycles from the chronopotentiometry experiments are shown here.

The PBA‐infiltrated electrodes and the use of nanopatterned IEMs demonstrated improved charge–discharge behavior and more efficient salt removal relative to the control cases with plain electrodes and flat IEMs, as shown by the cell voltage and NaCl effluent concentration profiles during cycling (**Figure** **8**a). The feed concentration was 35 000 ppm NaCl, and the cell was operated at a constant current density of 2 mA cm^−2^. The MCDI cell with a flat membrane exhibited a voltage ≈500 mV higher than the best‐performing configuration, which used a hexagonal nanopatterned membrane. PBA‐infiltrated and other patterned membranes showed intermediate voltages. This trend reflects the enhanced surface area achieved with hexagonal patterns. Figure 8b shows that constant current density leads to comparable amounts of salt removal with each configuration in MCDI.

To contextualize these findings, it is important to consider how constant current (CC) mode governs ion transport and electrochemical performance in MCDI systems. In CC mode, a fixed current is applied while the voltage adjusts in response to system resistance and ion movement. This approach allows precise control of charge and the salt removal rates. This is important for understanding how cell resistance governs energy use for a fixed salt removal rate. Similarly, Figure S11 (Supporting Information) presents voltage and effluent trends during cycling with a feed of 30 000 ppm NaCl and 5000 ppm MgSO_4_. The PBA‐infiltrated electrode exhibited a voltage ≈100 mV lower than with the 35 000 ppm NaCl feed. The voltage gap between the flat and hexagonal membranes remained ≈500 mV. Although concentration profiles were largely similar across configurations (Figure 7; Figure S12, Supporting Information), hexagonal nanopatterned membranes displayed more pronounced deviations. Extending the charge–discharge time from 120 to 210 s reduced concentration minima, as shown in SI 11. This suggests that patterned surfaces induce secondary flows that slow mass transfer but enhance ion selectivity. Tables S6 and S7 (Supporting Information) summarize the salt removal efficiency (SRE), average salt adsorption rate (ASAR), energy recovery (ER), energy normalized adsorbed salt (ENAS), and water recovery (WR) in one cycle for MCDI with flat, nanopatterned, and PBA‐infiltrated electrodes using 35 000 ppm NaCl and a mixture of 30 000 ppm NaCl and 5000 ppm MgSO_4_. The table indicates that ENAS increases for nanopatterned IEMs and PBA‐infiltrated electrodes, reaching maximum values of 382.10 and 423.40 mmol J^−1^, respectively, with concentric hexagonal patterns in both cases.

Here, the nanopatterned CEM and PBA electrode both exhibit selectivity toward Na⁺. In the CEM, the negatively charged sulfonic groups preferentially facilitate Na⁺ transport due to its smaller ionic radius and lower hydration energy. In the PBA electrode, Na⁺ undergoes rapid intercalation into the open cubic lattice, whereas the larger Mg^2^⁺ ions are largely excluded and adsorb only at the surface. For anions, the nanopatterned AEM enhances Cl^−^ transport over SO_4_^2^^−^ through charge‐based exclusion and ionic mobility differences. Additionally, the surface patterns improve local mixing and reduce boundary‐layer resistance, enhancing overall ion transport efficiency in the MCDI cell.

To evaluate the stability of the electrodes, we conducted 100 consecutive cyclic voltammetry scans using a 35 000 ppm NaCl solution. The current density from the long‐term cycle voltammograms of PBA analog coated electrode ≈+0.2 and −0.2 V is 45–50% higher than the current density of bare carbon cloth electrode at the same voltages (Figure S17, Supporting Information). The capacity retention (Q/Q_0_, where Q_0_ is the initial capacity) for the carbon cloth electrode (red rectangles) was calculated to be 82.7% after the 100th cycle, indicating excellent electrochemical stability. Similarly, the capacity retention for the PBA analog‐coated electrode was calculated to be 85.59% after the 100th cycle (Figure S18, Supporting Information). Also, the carbon cloth electrodes and PBA analog coated electrodes showed no visible structural or surface changes after cycling, confirming their robustness and suitability for long‐term CDI applications.

**Figure 9:**
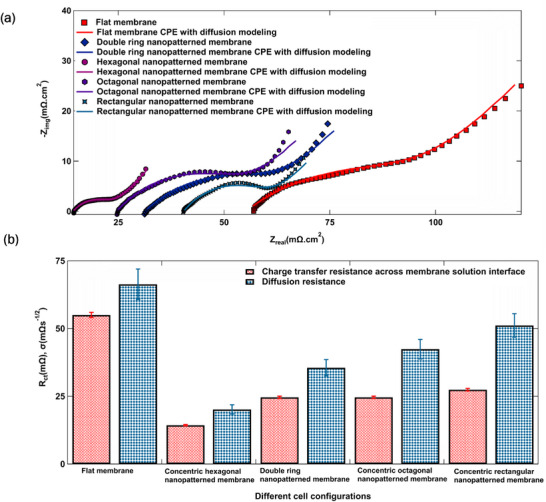
Nyquist plot from EIS and plot from equivalent circuit modeling (ECM) for a) flat, rectangular nanopatterned, double ring nanopatterned, octagonal nanopatterned, and hexagonal nanopatterned IEMs. b) charge‐transfer resistance) (R_ct_) values and diffusion resistance (σ) values extracted from the Nyquist plots.

MCDI cells with nanopatterned IEMs exhibited lower high‐frequency resistance (HFR) values relative to those with flat IEMs, with the hexagonally nanopatterned membrane achieving the most significant reduction—74% lower than the flat membrane configuration. Electrochemical impedance spectroscopy (EIS) was employed to probe the internal resistances of MCDI cells incorporating flat IEMs, nanopatterned IEMs, and PBA‐infiltrated electrodes. The corresponding Nyquist plots are shown in **Figure** **9**a, and the equivalent circuit and HFR values for each configuration are provided in Section S10 (Supporting Information). This trend is consistent with findings from constant current tests and surface area analyses. The semicircle diameter in the Nyquist plot, which reflects charge transfer resistance, is smallest for the hexagonal nanopatterned membranes. Figure 9b summarizes the charge‐transfer and diffusion resistances for all tested MCDI configurations. The semicircular response corresponds to a parallel resistor–capacitor element, representing ion accumulation and transport at the membrane–solution interface. These results suggest that hexagonal nanopatterned IEMs were more effective for removing ions from the membrane–liquid interface. Figure S15 (Supporting Information), depicting cell voltage vs transition time from the second chronopotentiometry graph, further confirms a stable voltage profile, indicating consistent electrode performance during ion adsorption and desorption.^[^ ^81^ ^]^

**Figure 10:**
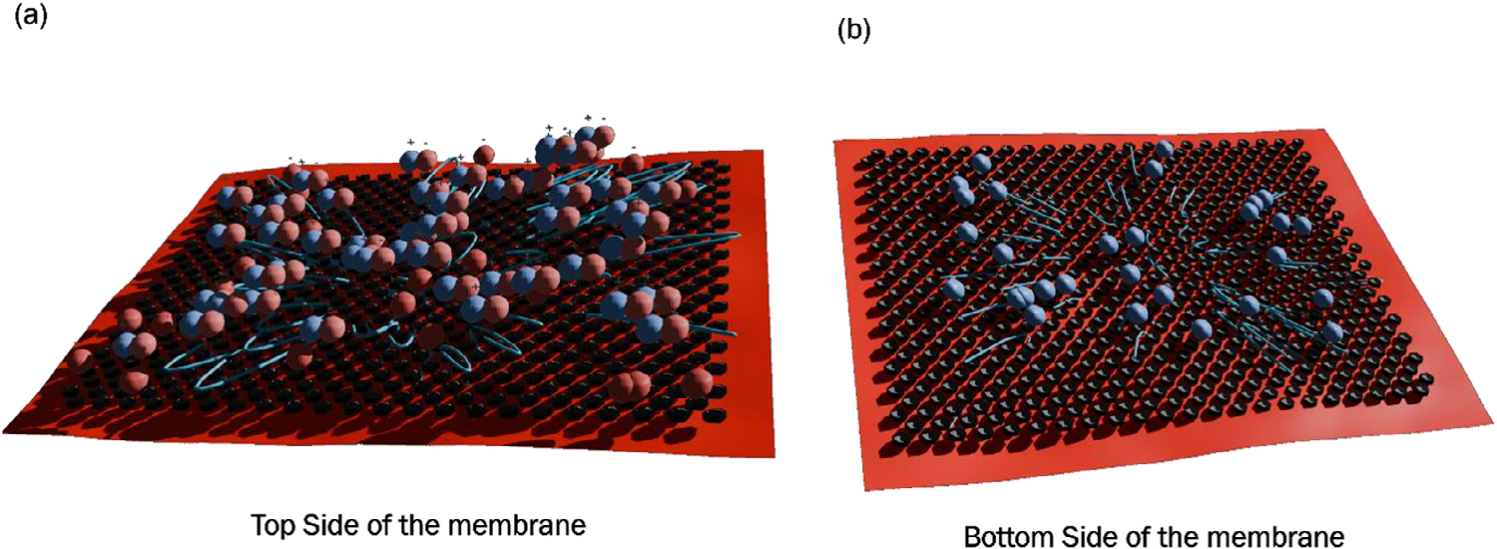
Possible interaction mechanism of ions with hexagonal nanopatterned membrane.

Based on the above results, we conclude that understanding ion transport at the nanoscale requires accounting for electrostatic interactions and the complex fluid dynamics induced by surface patterning. The schematic in **Figure** **10** illustrates ion transport through a nanopatterned cation‐exchange membrane featuring a hexagonal array of nanochannels. Secondary flows—visible as swirling eddies near the pore openings—arise from the membrane's surface topography and local charge distribution. These vortices enhance ion selectivity by possibly directing cations (Na⁺) toward the nanopores while displacing anions (i.e., Cl^−^) away from the membrane interface. Sodium ions are drawn into the negatively charged nanochannels, where confinement by nanopore geometry and partial dehydration (i.e., shedding part of the hydration shell) facilitate selective translocation. In contrast, Cl^−^ ions are electrostatically repelled and redirected by the circulating flow field, preventing their entry. The synergy between geometric confinement, charge‐based exclusion, and eddy‐enhanced transport results in highly efficient, directional ion separation across the membrane.

## 3 Conclusion

This work presents a novel and effective strategy to enhance membrane capacitive deionization (MCDI) performance for high‐salinity water treatment by integrating three key innovations: nanopatterned ion‐exchange membranes, dilute regeneration solutions, and Prussian blue analog (PBA)‐modified electrodes. Dilute regeneration solutions markedly improve electrode recovery, while Prussian blue analog (PBA) modification enhances ion storage by introducing redox‐active sites. Among the surface geometries tested, hexagonal nanopatterns provided the greatest surface area enhancement and most significant improvements in ion transport. We hypothesize that these patterns not only increase membrane–solution contact area but also induce micro‐scale fluid vortices that guide sodium ions into the pores while deflecting unwanted anions—substantially improving selectivity.

The narrow nanochannels further facilitated efficient Na⁺ transport by promoting partial dehydration and confinement‐driven ion mobility.^[^ ^82^ ^]^ In parallel, PBA‐functionalized electrodes enhanced salt adsorption and charge transfer, reducing overall resistance and enabling stable performance over repeated cycles. Together, these design elements resulted in lower cell voltage, higher salt removal efficiency, and improved system recovery, even when desalinating seawater‐strength or mixed‐salt solutions.

Our results reveal that integrating nanopatterned IEMs with PBA‐infiltrated electrodes significantly improves desalination performance. Among the configurations tested, hexagonal nanopatterned membranes achieved the greatest surface area enhancement, resulting in a 500 mV reduction in cell voltage, a 45 Ω cm^2^ decrease in area‐specific resistance, and a 19 Ω cm^2^ drop in charge transfer resistance. These results underscore the synergistic effect of membrane patterning and redox‐active electrode materials in reducing energy demands during MCDI operation.

Moreover, the system maintained stable performance across a broad range of regeneration solution concentrations (0–5000 ppm NaCl), with minimal ion accumulation even under highly saline conditions. The use of dilute regeneration solutions–specifically 2000 ppm NaCl and mixed 2000 ppm NaCl–MgSO_4_ solutions–enabled effective and energy‐efficient electrode recovery, emphasizing the practical feasibility of this approach.

Our work shows that low‐concentration regeneration solutions can fully restore electrode capacity without compromising performance, offering a pathway toward more sustainable and energy‐efficient operation. Overall, the synergy between patterned membrane design, functionalized electrodes, and smart regeneration unlocks new opportunities for scalable, low‐energy desalination technologies capable of addressing real‐world water challenges.

## 4 Experimental Section

### Materials

Biphenyl, N,N‐dimethylacetamide (DMAc), sodium chloride (NaCl), tetrahydrofuran (THF), trimethylamine (33% in ethanol), trifluoromethanesulfonic acid, potassium thioacetate, hydrogen peroxide, formic acid, and sulfuric acid were purchased from Sigma–Aldrich and used as received. Carbon black (Vulcan XC72R) was obtained from Cabot Corporation, and polyvinylidene fluoride (PVDF) was purchased from Arkema High Performance Polymers. 7‐Bromo‐1,1,1‐trifluorohexan‐2‐one and m‐terphenyl were sourced from SyQuest Inc. (USA). Dimethyl sulfoxide, dichloromethane, methanol, and acetone were sourced from VWR. Carbon cloth was acquired from Kuraray and activated by treatment with 1 m nitric acid at 85 °C prior to use as carbon electrodes. Photoresist and Sylgard 184 silicone elastomer were supplied by the Penn State Nanofabrication Facility, where the complete nanopatterning process was performed.

### Fabrication of Nanopatterned IEMs

Initially, 4‐inch silicon wafers underwent spin coating at a rotational speed of 3000 rpm for a duration of 45 s to deposit the e‐beam resist. The coating solution (i.e., e‐beam resist) consisted of Zep 520A121 positive resist and anisole, with a mixture ratio of 1:1. Subsequently, the silicon wafer was baked at 180°C for a duration of 2 min. Subsequently, it was exposed to the RAITH EBPG 5200 electron beam lithography system at an electron beam current of 150 nanocoulombs per square centimeter of the silicon wafer, through an aperture of 600 micrometers. The pattern was developed by immersing the silicon wafer in n‐Amyl acetate (pentyl acetate) for a duration of 3 min and in 2‐propanol for a duration of 1 min. Finally, the silicon wafer was dried using nitrogen gas.

In the next step, polydimethylsiloxane (PDMS) was cast and cured on the patterned silicon master. PDMS is a biocompatible, chemically inert, thermally stable, and optically transparent elastomer that is widely used in microfluidics and lab‐on‐a‐chip technologies.^[^ ^83^, ^84^, ^85^, ^86^ ^]^ Its flexibility and robustness also make it ideal as a reusable submaster mold for repeated drop‐casting of ionomer solutions to fabricate ion‐exchange membranes (IEMs).

To prepare the PDMS mold, Sylgard 184 silicone elastomer base and curing agent were mixed at a 10:1 volume ratio. The components were thoroughly stirred in a plastic cup for 7–10 min until a uniform, milky consistency was achieved. Inadequate mixing could result in unreacted curing agents, compromising mechanical integrity and bonding. The mixing process introduced air bubbles, which were removed by vacuum degassing in a desiccator at a residual pressure of 10–20 inches of mercury for 30 min.

The degassed PDMS mixture was then gently poured over the silicon wafer (patterned side facing up), placed in a plastic Petri dish, taking care to minimize bubble formation. The entire assembly was cured in an oven at 65 °C for 4 h. After curing, the Petri dish was carefully cut open using wire clippers to expose the mold. Finally, the PDMS was gently separated from the silicon master by slowly peeling it away, preserving the fine structural details of the patterned surface.

Nanopatterned poly(phenyl alkylene) AEMs and CEMs were fabricated by drop‐casting 5 wt.% solutions of either poly(terphenylene alkylene) tethered with trimethylammonium groups or poly(biphenylene) with pendant sulfonic acid groups onto nanopatterned PDMS submaster molds. The assemblies were placed in an oven at 60 °C and allowed to dry for 18 h to ensure complete solvent evaporation. Following solvent evaporation, the resulting nanopatterned ion‐exchange membranes (IEMs) were carefully peeled from the PDMS mold, preserving the high‐fidelity surface features imparted by the nanopatterned template. Detailed procedures for polymer synthesis and characterization are provided in Sections S1, S2, S4, and S5 (Supporting Information).

### Preparation of PBA Analog Infiltrated Electrodes

PBAs were synthesized via co‐precipitation. A 0.2 m metal nitrate solution (M(NO_3_)_2_, M ═ Co^2^⁺, Ni^2^⁺, or Cu^2^⁺) was added dropwise to a mixture containing 0.1 m Na_4_Fe(CN)_6_, 0.2 m sodium citrate, and 1 m NaNO_3_ under vigorous stirring. Sodium citrate acted as a chelating agent to control nucleation, and NaNO_3_ provided a Na⁺‐rich medium for structural incorporation. The suspension was aged for 24 h, centrifuged, washed with ethanol and deionized water, then vacuum‐dried at 60 °C for 8 h and ground to a fine powder. Then, Nickel PBA (NaNi[Fe(CN)_6_]·nH_2_O) powder was mixed with PVDF and conductive carbon (8:1:1) in 1‐methyl‐2‐pyrrolidone to prepare a coating ink, which was then spray‐painted onto activated carbon cloth electrodes. The weights of nickel PBA, PVDF, and carbon black used to prepare the ink were 0.344, 0.043, and 0.043 g, respectively, yielding a total coating weight of 0.43 g. After that, the electrodes were dried in an oven at 60 °C for 2 h. Both blank activated carbon cloth electrodes and PBA analog‐infiltrated electrodes were examined by SEM and EDS to confirm the presence of the PBA analog within the porous structure.

### X‐Ray Photoelectron Spectroscopy

XPS experiments were performed using a Physical Electronics Versa Probe III instrument equipped with a monochromatic Al kα X‐ray source (hν = 1486.6 eV) and a concentric hemispherical analyzer. Charge neutralization was performed using both low‐energy electrons (<5 eV) and argon ions. The binding energy axis was calibrated using sputter‐cleaned Cu (Cu 2p_3/2_ = 932.62 eV, Cu 3p_3/2_ = 75.1 eV) and Au foils (Au 4f_7/2_ = 83.96 eV).^[^ ^87^ ^]^ Peaks were charge referenced to the CH_x_ band in the carbon 1s spectra at 284.8 eV. Measurements were made at a takeoff angle of 45° with respect to the sample surface plane. This resulted in a typical sampling depth of 3–6 nm (95% of the signal originated from this depth or shallower). Quantification was done using instrumental relative sensitivity factors (RSFs) that account for the X‐ray cross‐section and inelastic mean free path of the electrons. The analysis size was ≈200 µm in diameter.

### Atomic Force Microscopy

The peak force tapping scans were performed on a Dimension Icon AFM (Bruker) with ScanAsyst Air (Bruker) probes with a nominal tip radius of 2 nm and a spring constant of 0.4 N m^−1^. Scans of 20 and 5 µm with a probe vibration frequency of 2 kHz, peak force amplitude of 150 nm, 512 samples/line, and a scan rate of 0.35 Hz was performed. Images and roughness were analyzed with NanoScope Analysis v.3.

### Flow‐by MCDI

A commercial membrane capacitive deionization (MCDI) cell (Ecscell) with an active membrane area of 25 cm^2^ (5 cm × 5 cm) was employed for deionization experiments. Cell voltage was precisely regulated using a Gamry potentiostat, and conductivity was continuously monitored with an in‐line conductivity probe (Microelectrodes Inc.). The feed solution was circulated through the cell using a peristaltic pump (ANKO). To establish operating conditions, five cycles of chronoamperometry were first performed with an upper voltage limit of +1.5 V and a lower voltage limit of −1.5 V, selected to minimize parasitic side reactions at the porous carbon electrodes.^[^ ^88^ ^]^ The average current obtained from these cycles was subsequently applied during chronopotentiometry experiments. Additional details of the MCDI setup are provided in Section S8 (Supporting Information).

### Calibration for Ionic Conductivity Probes

Ionic conductivity probes were obtained from Microelectrodes Inc. and installed at the outlet of the MCDI device. To determine the cell constant, a 0.01 N standard KCl solution was passed through the setup, yielding a value of K ≈ 0.99 cm^−1^, in accordance with the manufacturer's instructions. Subsequently, conductivity measurements were performed using NaCl solutions with concentrations ranging from 0 to 35 000 ppm. The resulting calibration curve, showing a linear relationship between conductivity and concentration, is provided in Section S9 (Supporting Information). Following each deionization experiment, the conductivity probe was soaked in deionized water for 24 h to ensure complete wetting and reliable performance in subsequent measurements.

### Electrochemical Impedance Spectroscopy (EIS)

Potentiostatic EIS measurements were conducted using a Gamry Reference 3000 potentiostat. A small sinusoidal voltage perturbation of 10 mV was applied without any background (DC) bias. The frequency was swept from 100 kHz to 0.1 Hz, with 10 data points collected per decade to ensure high‐resolution spectra.

## Conflict of Interest

The authors declare no conflict of interest.

## Supporting information



Supporting Information

## Data Availability

The data that support the findings of this study are available in the supplementary material of this article.
