# Supplementary material for: Dilute Regeneration‐Driven Membrane Capacitive Deionization of Synthetic Seawater using Nanopatterned Membranes and Prussian Blue Analog Electrodes
Source: Small. 2025 Nov 24;22(3):e10773. doi: 10.1002/smll.202510773 (PMC12802532; doi:10.1002/smll.202510773)
Supplement: Supplementary file 1 — Supporting Information [file SMLL-22-e10773-s001.pdf]

NANO · MICRO
small

Supporting Information

for *Small*, DOI 10.1002/smll.202510773

Dilute Regeneration-Driven Membrane Capacitive Deionization of Synthetic Seawater using Nanopatterned Membranes and Prussian Blue Analog Electrodes

*Mahmudul Hasan, Michael Labella, Colton Waters Burke, Christopher G. Arges, Enrique D. Gomez and Christopher A. Gorski**

Supporting Information

for

Dilute Regeneration-Driven Membrane Capacitive Deionization of Synthetic Seawater Using Nanopatterned Membranes and Prussian Blue Analog Electrodes

Mahmudul Hasan[a,b], Michael Labella[c], Colton Waters Burke[a], Christopher G. Arges[a,d], Enrique D. Gomez[a,c,e], Christopher A. Gorski[b,*]

[a]Department of Chemical Engineering, The Pennsylvania State University, University Park, PA-16802

[b]Department of Civil and Environmental Engineering, The Pennsylvania State University, University Park, PA-16802

[c]Materials Research Institute, The Pennsylvania State University, University Park, PA-16802

[d]Argonne National Laboratory, 9700 S. Cass Avenue, Lemont, IL 60439

[e]Department of Materials Science and Engineering, The Pennsylvania State University, University Park, PA 16802

[*]Corresponding author: Christopher A. Gorski, Email address: cag981@psu.edu

Section S1. Synthesis of AEM

The synthesis protocol used for the anion exchange membrane is depicted in Figure S1.

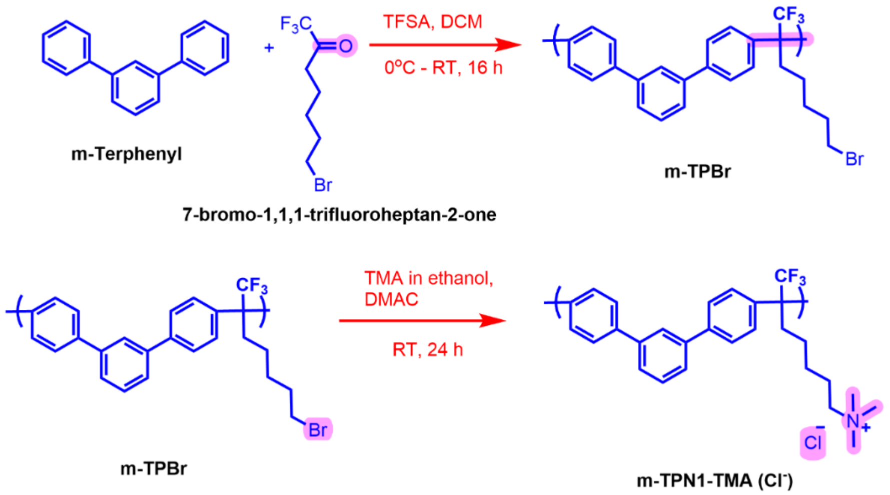

Figure S1: Synthesis of anion exchange membrane (mTPN1)

Step-1: Synthesis of mTPBr

A 3-neck 250 mL round bottom flask equipped with a nitrogen inlet, magnetic stirrer, and dropping funnel. To this, m-terphenyl (10 g, 43.42 mmol) and 7-bromo-1,1,1-trifluorohexane-2-one (11.80 g, 47.75 mmol) were added. Purge nitrogen gas for 30 minutes and 60 mL of anhydrous DCM was added. The reaction flask was cooled to 0 °C using an ice bath. To this, trifluoromethanesulfonic acid (31 mL, 347 mmol) was added dropwise using a dropping funnel. After complete addition of trifluoromethanesulfonic acid, remove the ice bath and stir the reaction mixture at room temperature for 16 hours. The viscous solution was poured into methanol (600 mL), washed with methanol several times, and dried. The dried polymer was then redissolved in 200 mL THF, precipitated in methanol (1000 mL), filtered, and dried in a vacuum oven overnight.

Step-2: Synthesis of mTPN1

Into a 250 mL round bottom flask equipped with a stirring bar, mTPBr (15 g, 47.1 mmol), DMAc (150 mL) and trimethylamine (33 w/w% in EtOH, 34 mL, 141 mmol) were added. The reaction mixture was stirred at room temperature for 24 hours. The reaction mixture was precipitated dropwise into 1000 mL THF. After precipitation, the THF solution was replaced with fresh acetone (500 mL). The polymer was filtered, collected by filtration, and then dried in a vacuum oven at 60 °C overnight.

Step-3: Preparation of AEM Membrane

The AEM membrane was prepared by drop casting of polymer in DMAc (~5 wt %, w/v) onto a clean and leveled glass plate. The solvent was slowly evaporated at 60 °C for 16 hours and at 120 °C for 2 hours. The obtained membrane was removed from the petri dish, immersed in DI water, and dried in a vacuum oven at 50 °C for 24 hours. The membrane thickness was measured by a digital micrometer, and it was 45 ± 5 microns.

Section S2. Synthesis of CEM

The synthesis protocol used for the cation exchange membrane is depicted in Figure S2.

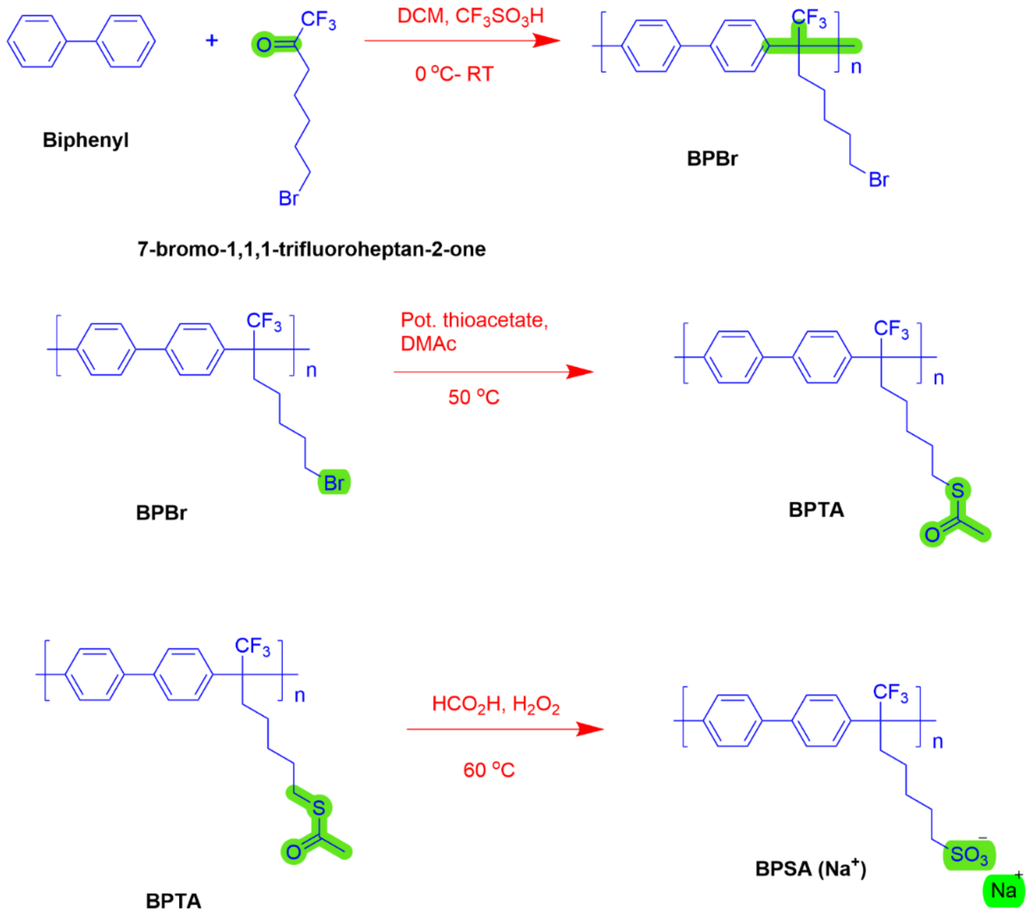

Figure S2: Synthesis of cation exchange membrane (BPSA)

Step-1: Synthesis of BPBr

A three-neck 250 mL size round bottom flask equipped with a nitrogen inlet, magnetic stirrer, and additional funnel. To this, biphenyl (10 g, 64.85 mmol) and 7-bromo-1,1,1-trifluorohexane-2-one (17.62 g, 71.33 mmol,) were added. After N_2 purging for 30 minutes, 70 mL of anhydrous

DCM was added. The reaction flask was cooled to 0 °C in the ice bath. Trifluoromethanesulfonic acid (35 mL, 389 mmol) was added dropwise using a dropping funnel. After complete addition of trifluoromethanesulfonic acid, remove the ice bath and stir at room temperature for 14 hours under nitrogen. The viscous reaction mixture was precipitated into methanol (600 mL) as the fibrous shape, washed with methanol, filtered, and dried. The dried polymer was then redissolved in 300 mL THF, precipitated in methanol (1500 mL), filtered, and dried in a vacuum oven overnight (50 °C).

Step-2: Synthesis of BPTA

To a 100 mL two-neck round bottom flask equipped with a stirring bar and nitrogen inlet. BPBr (4 g, 10 mmol), potassium thioacetate (2.35 g, 20 mmol), and DMAc (40 mL) were added. The solution was then heated to 60 °C in an oil bath for 6 hours. The reaction mixture was cooled to room temperature and precipitated into 200 mL of methanol. The crude polymer was collected by filtration and dried. The crude polymer was dissolved into THF (40 mL), precipitated into methanol (200 mL), filtered, and dried in a vacuum oven at 50 °C for 24 hours.

Step-3: Heterogenous oxidation of BPTA to BPSA

The BPTA membrane was prepared by solution casting into DMAc at a 5% wt/v concentration. The solutions were then passed through a short plug of cotton to remove any dust particles. The freshly filtered solution was then poured onto a clean and leveled glass plate and heated at 60 °C for 16 hours and at 120 °C for 2 hours. The membrane was removed from the glass plates by immersing in deionized water and then dried.

The BPTA membrane was placed into a solution of 30 % hydrogen peroxide, formic acid, and water in a 1:1:4 ratio by volume (10 mL:10 mL:40 mL) and heated to 60 °C for 6 h. The membrane was rinsed thoroughly with water, then placed into 1 M H_2SO_4 for 24 h. Then, the membranes were rinsed with deionized water until the rinse solution became neutral. The membranes were then placed into fresh deionized water for 24 h, replaced with fresh water three times in 24 hours.

Step-4: Oxidation of BPTA to BPSA

To a 100 mL two-neck round bottom flask equipped with a stirring bar and nitrogen inlet. BPBr (2 g, 5.2 mmol), potassium thioacetate (0.66 g, 5.7 mmol), and DMAc (40 mL) were added. The solution was then heated to 60 °C in an oil bath for 6 hours. The reaction mixture was cooled to 0 °C in an ice bath. m-CPBA (2.7 g, 15.6 mmol) dissolved in 5 mL DMAc was added dropwise over 30 minutes by dropping funnel and stirred at room temperature for 1 hour. The reaction mixture was precipitated into 400 mL 1M NaCl and stirred for 1 hour. The polymer was collected by filtration and dried. The crude polymer was redissolved into DMSO (40 mL), precipitated into acetone (200 mL), filtered, and dried in a vacuum oven overnight.

Section S3. AFM maps of nanopatterns of different geometric shapes

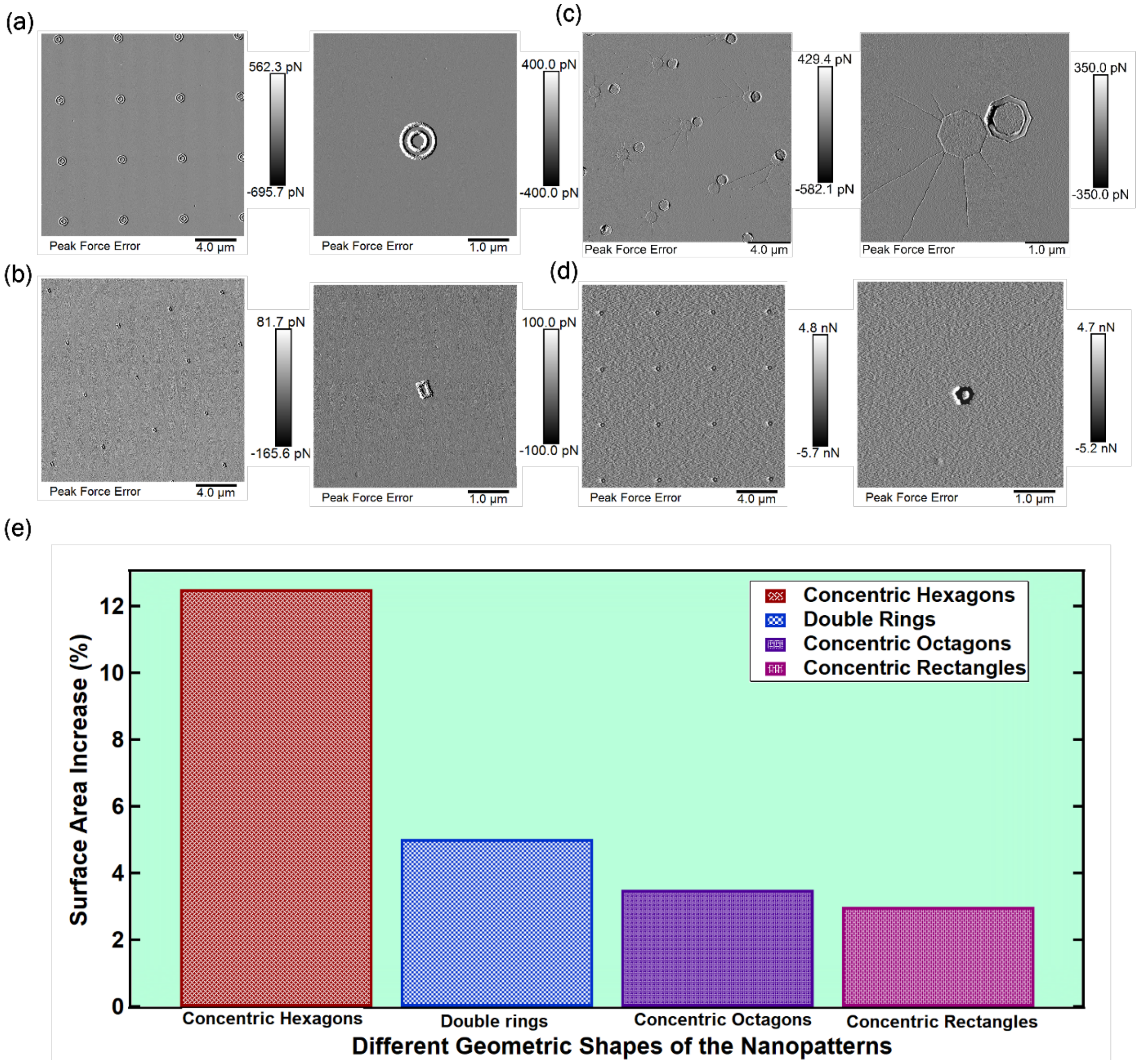

Figure S3. Topographical AFM maps of the a) double ring, b) concentric rectangular, c) concentric octagonal and d) concentric hexagonal nanopatterned IEMs. (e) Percentage surface area increase of IEMs due to the contribution of a single nanopattern across different geometric shapes.

Section S4. NMR spectrums of AEM and CEM preparation

Structural characterization of polymers was performed using ^1H NMR spectroscopy. The ^1H NMR spectra were recorded on Bruker 400 MHz in $CDCl_3$ or DMSO-d6. Molecular weights were determined by Gel Permeation Chromatography (GPC) in THF using polystyrene as a standard. ^1H NMR spectra of the polymers are shown here.

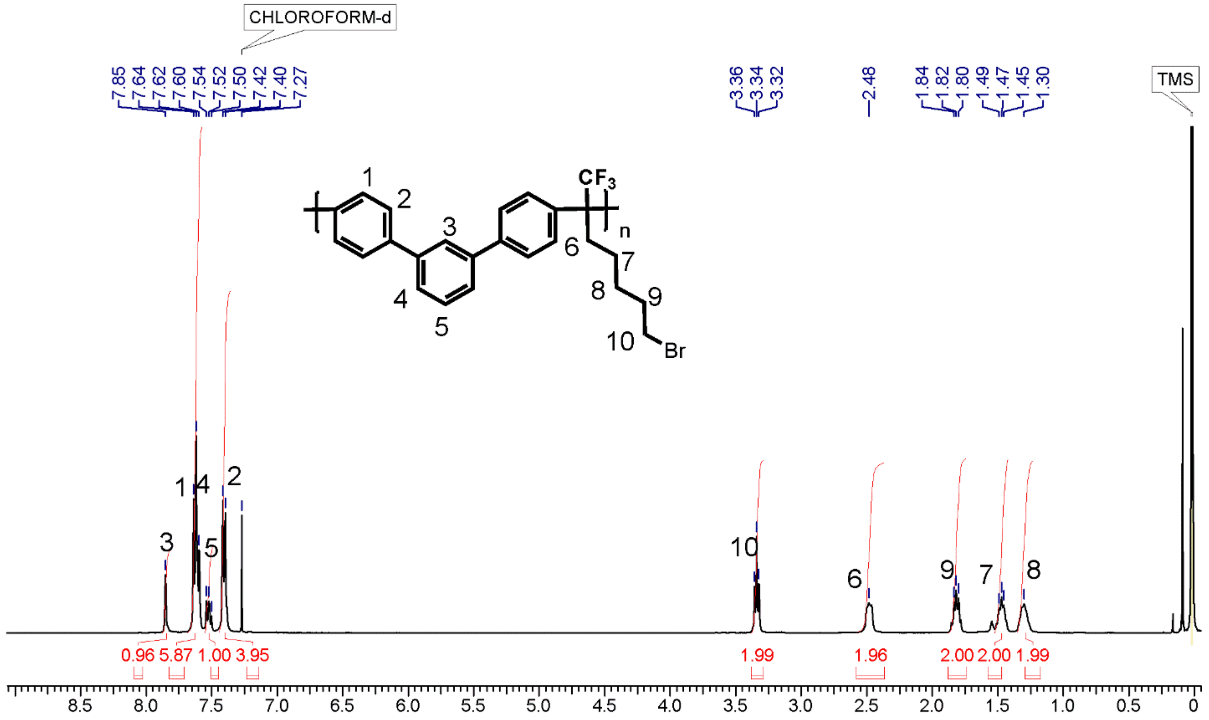

Figure S4: ^1H NMR spectrum of mTPBr in $CDCl_3$.

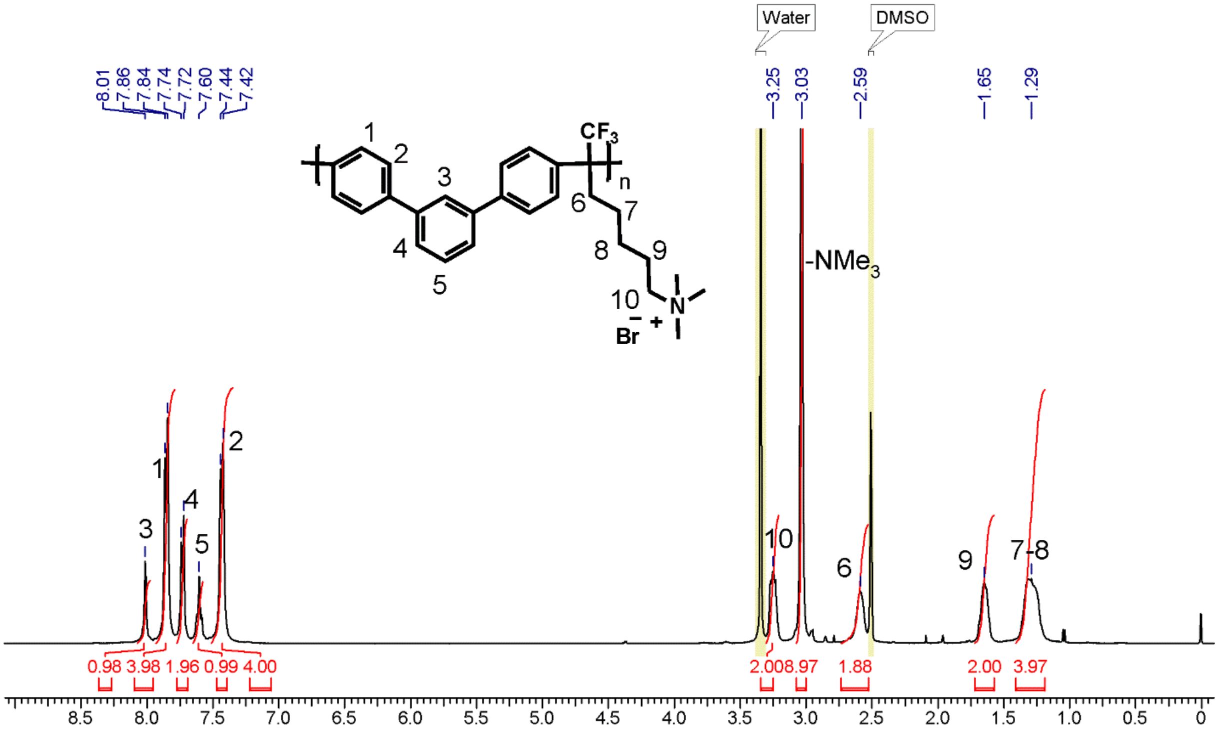

Figure S5: ^{1}H NMR spectrum of mTPN1 in DMSO-d6.

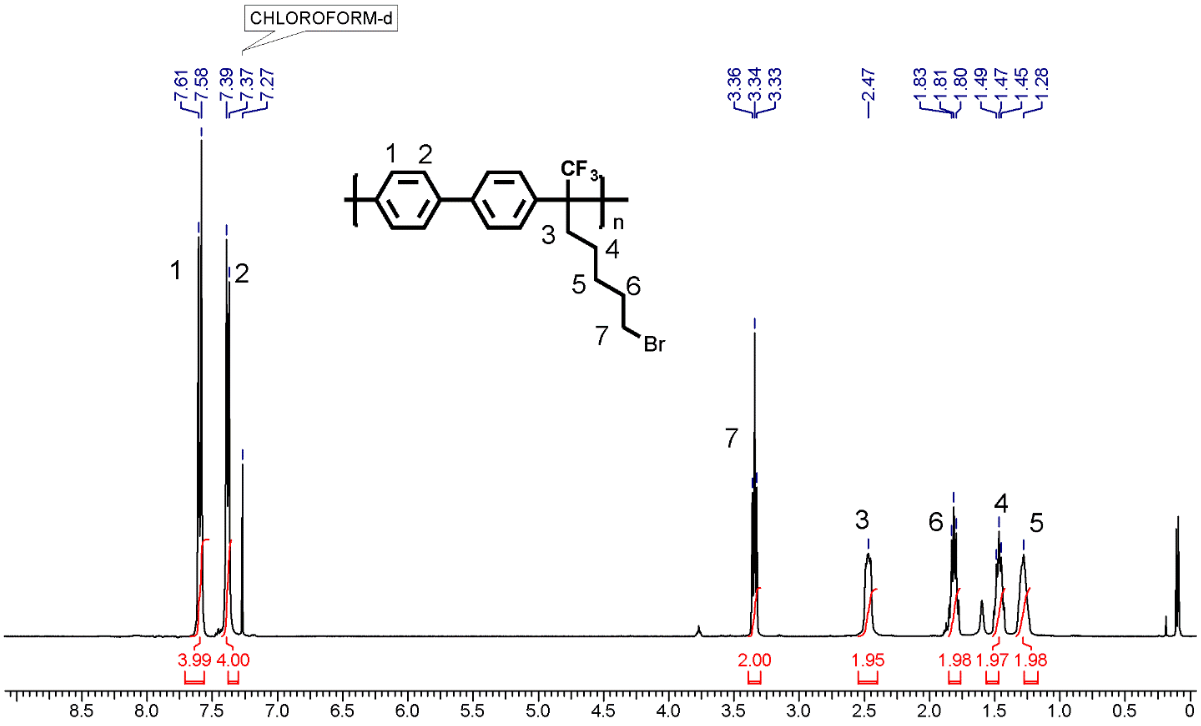

Figure S6: ^{1}H NMR spectrum of BPBr in $CDCl_3$.

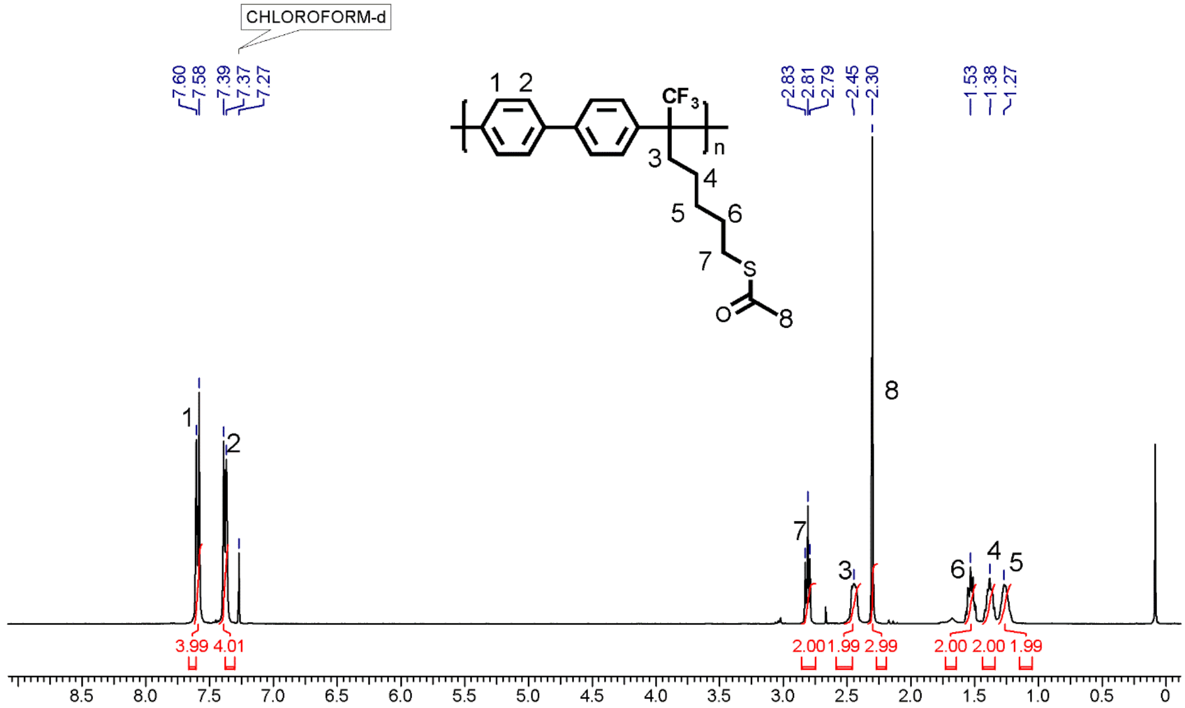

Figure S7: ^{1}H NMR spectrum of BPTA in $CDCl_3$.

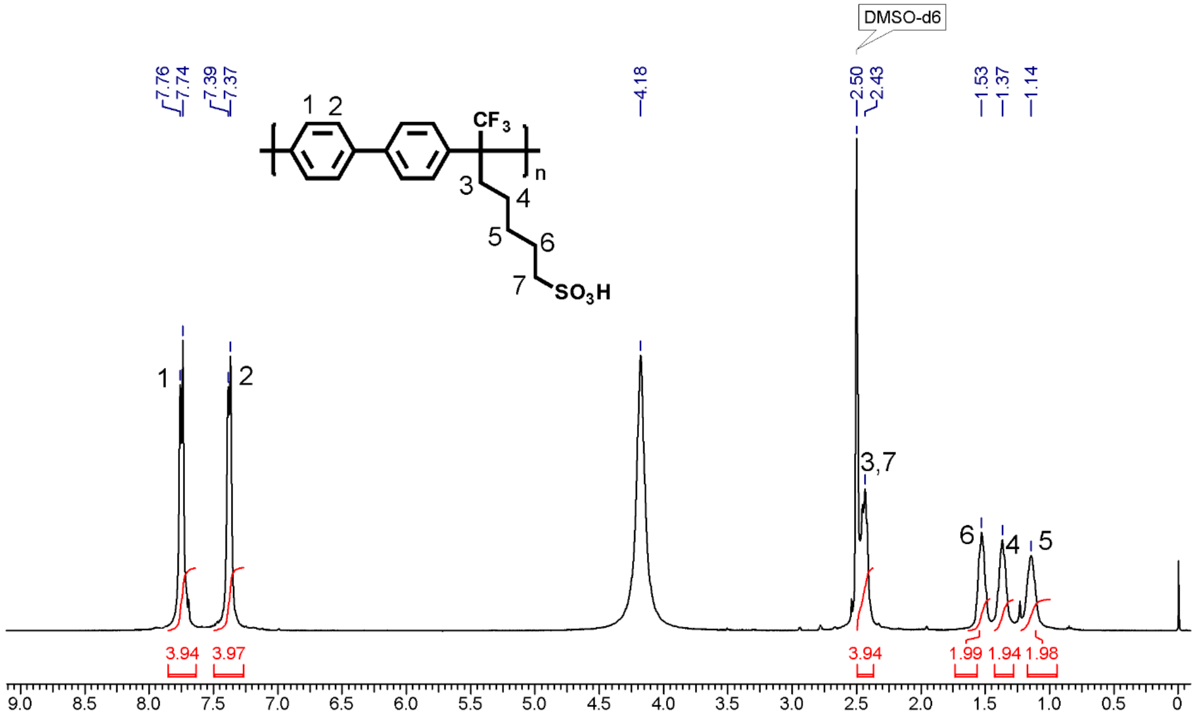

Figure S8: ^{1}H NMR spectrum of BPSA in $CDCl_3$.

Section S5. Different properties of ion exchange membranes

IEC measurements

AEM

The IEC of AEM membrane was determined by ^{1}H NMR spectroscopy and Mohr titration. A dry membrane in chloride form (100 mg) was immersed in 50 mL of a 0.1 M sodium nitrate solution for 24 hours. The sodium nitrate solution was transferred to the 250 mL beaker and the membrane was washed three times with 0.1 M sodium nitrate solution. 3-4 drops of potassium chromate were added (indicator) to the solution and titrated with 0.1 M silver nitrate solution. The IEC was calculated using the following equation.

$$\text{IEC (mmol/g)} = \frac{V_{\text{silver nitrate}} \times C_{\text{Silver nitrate}}}{W_d} \qquad \text{(Eq. S1)}$$

Where $V_{\text{silver nitrate}}$ is the amount of silver nitrate added (in mL), $C_{\text{silver nitrate}}$ is the concentration of silver nitrate solution and W_d is the dry mass of the membrane.

CEM

Dried 100 mg BPSA membrane (in –SO_3H form) was immersed in 1 M NaCl (50 mL) for 24 hours. The solution was transferred to the 250 mL beaker. The membrane was washed three times with 10 mL 1M NaCl. Add 3-4 drops of phenolphthalein in it and the NaCl solution was titrated with 0.01 m NaOH. The IEC was measured using the following equation.

$$\text{IEC (mmol/g)} = \frac{V_{\text{NaOH}} \times C_{\text{NaOH}}}{W_d} \qquad \text{(Eq. S2)}$$

Where V_{NaOH} is the amount of NaOH added (in mL), C_{NaOH} is the concentration of standardized NaOH solution and W_d is the dry mass of the membrane.

Water uptake

100 mg of dry membranes (AEM in chloride form and CEM in sodium form) were immersed in DI water for 24 hours at 30 °C. The fully hydrated membranes were removed from the water and removed the surface water using Kim wipe and then weight immediately. An average of three independent measurements is reported. The water uptake was calculated using the following equation.

$$\text{Water uptake (\%)} = \frac{W_w - W_d}{W_d} \times 100 \qquad \text{(Eq. S3)}$$

where W_d and W_w are the dry and wet masses of membrane, respectively.

Swelling ratio

The squared cut 3 cm*3 cm dry membranes were soaked in DI water for 24 hours at 30 °C. The hydrated membranes were removed the from water bath and blotted with Kim wipe to remove surface water and measure dimensions immediately. The swelling ratio was calculated using the following equation.

$$\text{Swelling ratio (\%)} = \frac{L_w - L_d}{L_d} \times 100 \qquad \text{(Eq. S4)}$$

where L_d and L_w are the dry and wet lengths of membrane, respectively.

Table S1. Properties of Ion Exchange Membranes

Membrane type	Thickness (μm)	IEC (mequiv g^{-1})	WU (%)	SR (%)	K (mS/cm) in DI water
BPSA CEM	30	2.4	26	9	29.8
m-TPN1 AEM	41	2.1	20	5	14.7
Concentric hexagonal BPSA CEM	29	2.6	28	11	31.3
Concentric hexagonal m-TPN1 AEM	38	2.3	23	7	15.5
Concentric octagonal BPSA CEM	27	2.5	29	9	30.7
Concentric octagonal m-TPN1 AEM	35	2.3	22	6	15.1
Double ring BPSA CEM	25	2.6	27	10	30.4
Double ring m-TPN1 AEM	34	2.2	21	6	14.9
Concentric rectangular BPSA CEM	28	2.5	26	8	30.2
Concentric rectangular m-TPN1 AEM	33	2.1	23	5	14.8

Section S6. SEM and FIB cross-sectional SEM

Scanning electron microscopy (SEM) images were collected using a FEI Helios 660 NanoLab focused ion beam (FIB) system. Before analysis, the surfaces were coated in a thin (10 nm), amorphous carbon layer to ensure high-fidelity measurements with the SEM. This was done using a Leica EM ACE600 Sputter Coater. The accelerating voltage used to collect SEM images was 5kV with currents ranged from 0.40 - 0.80 nA.

Cross-sectional scanning electron microscopy (SEM) samples were sectioned and imaged using a FEI Helios 660 NanoLab focused ion beam (FIB) system. Before analysis, the surfaces were coated in a thin (10 nm), amorphous carbon layer to ensure high-fidelity measurements in the SEM and high-resolution milling with the Ga+ beam. This was done using a Leica EM ACE600 Sputter Coater. In the FIB, Ga^+ ions (30 kV accelerating voltage, currents ranging from 2.5 nA to 0.23 nA) were used to selectively cross section the regions of interest. The accelerating voltage/current used to collect SEM images was 5kV, the currents ranged from 0.40 - 0.80 nA.

Section S7. Repetitive chronopotentiometry experiment for mixture of 30000 ppm NaCl and 5000 ppm $MgSO_4$

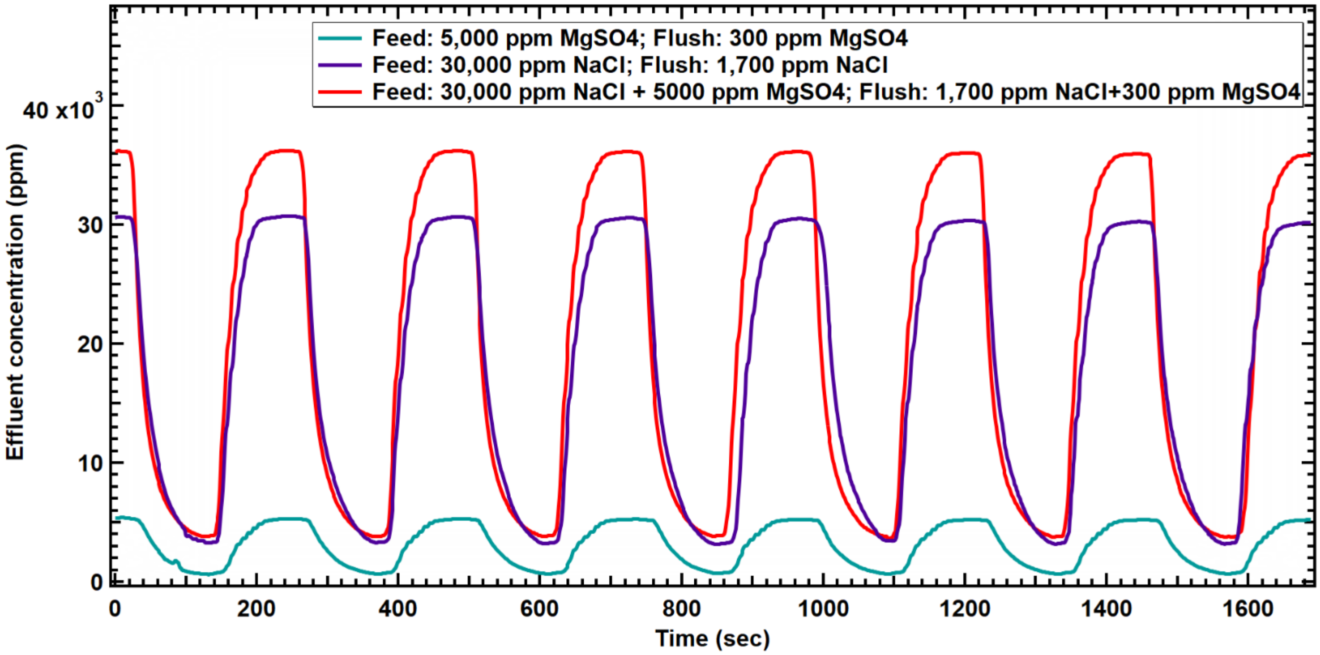

Figure S9. Effluent concentration profiles in MCDI for a 30,000 ppm NaCl and 5,000 ppm $MgSO_4$ mixture flushed with 1,700 ppm NaCl and 300 ppm $MgSO_4$ (red), 5,000 ppm $MgSO_4$ flushed with 300 ppm $MgSO_4$ (green), and 30,000 ppm NaCl flushed with 1,700 ppm NaCl (purple), using flat poly(phenylene alkylene) IEMs and porous carbon cloth electrodes at a current density of 2 mA cm^{-2}. Both the charge (deionization) and discharge (regeneration) steps were 120 seconds each. The deionization profile of the NaCl-$MgSO_4$ mixture (red) closely resembles the sum of the individual profiles for NaCl (purple) and $MgSO_4$ (green).

Section S8. MCDI experimental setup and calculations

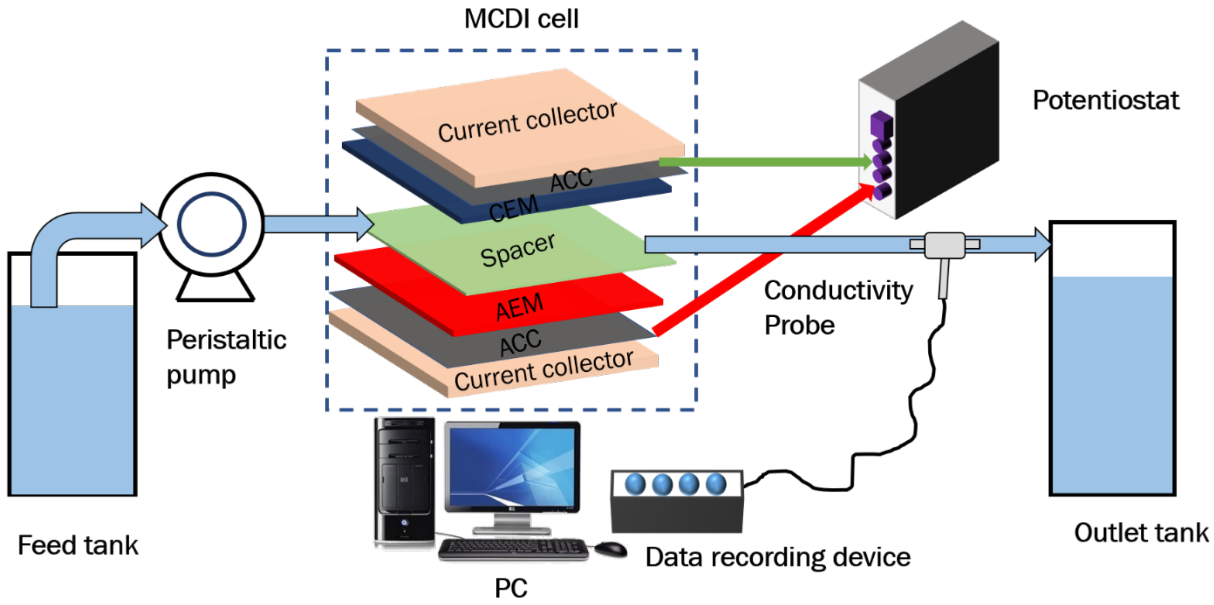

Figure S10: Different components of the membrane capacitive deionization along with inlet, outlet, Potentiostat, in line conductivity probe and data recording device

The parameters used to assess MCDI performance, and their corresponding equations, are given below.

The salt removal efficiency (SRE) is the amount of salt removed during the charging cycle and is calculated from equation S5.

$$\text{SRE}\ (\%) = \left(1 - \frac{C_{eff}}{C_0}\right) \times 100\% \qquad \text{(Eq. S5)}$$

Here, C_{eff} and C_0 correspond to the effluent and feed stream salt concentrations, respectively.

The average salt absorption rate (ASAR) is the average amount of ions absorbed by the electrode per area of electrode duration of charge cycle and is calculated from equation S6.

$$\text{ASAR}\ (\text{mol m}^{-2}\text{s}^{-1}) = \frac{\emptyset}{A \times t_{charge}} \int_0^{t_{charge}} (C_0 - C_{eff})\, dt \qquad \text{(Eq. S6)}$$

Here, $\emptyset$ is the volumetric flow rate of the process stream, and t_{charge}= charge cycle time.

A is the area of the geometric area of the cell for salt flux (25 cm^2).

The energy normalized absorbed salt (ENAS) is calculated from equation S7. This value corresponds to the amount of salt removed per unit of energy.

$$\text{ENAS (mol J}^{-1}) = \frac{\emptyset \int_0^{t_{charge}} (C_0 - C_{eff})dt}{E_{charge} - E_{discharge}} \qquad \text{(Eq. S7)}$$

E_{charge} is the amount of energy consumed to remove one ion during the charging cycle and is calculated from equation S8.

$$E_{charge}\ (\text{kT ion}^{-1}) = \frac{I_{charge} \int_0^{t_{charge}} V_{charge} dt}{2\emptyset \times N_A \times 4.11 \times 10^{-21} \times \int (C_0 - C_{eff})\, dt} \qquad \text{(Eq. S8)}$$

$E_{discharge}$ is the amount of energy consumed during the discharge cycle and is calculated from equation S9.

$$E_{discharge}\ (\text{kT ion}^{-1}) = \frac{I_{discharge} \int_0^{t_{discharge}} V_{discharge} dt}{2\emptyset \times N_A \times 4.11 \times 10^{-21} \times \int (C_0 - C_{eff})\, dt} \qquad \text{(Eq. S9)}$$

where $t_{disharge}$= discharge cycle time

Energy recovery (ER) is the amount of energy recovered during the charging cycle and is calculated from equation S10.

$$\text{ER (\%)} = \frac{E_{discharge}}{E_{charge}} \times 100 \qquad \text{(Eq. S10)}$$

Section S9. Calibration curve of ionic conductivity probe

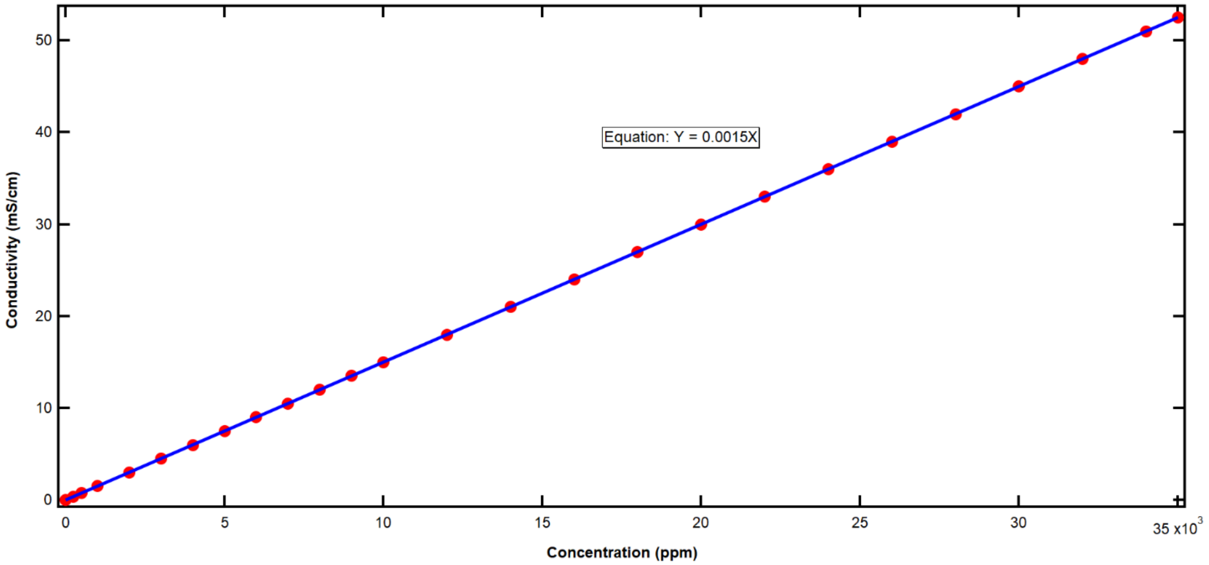

Figure S11: Conductivity vs concentration plot for the ionic conductivity probe across different concentrations ranging from 0 to 35000 ppm NaCl

Section S10. Repetitive chronopotentiometry data with different cell configurations for a mixture of 30,000 ppm NaCl and 5,000 ppm $MgSO_4$.

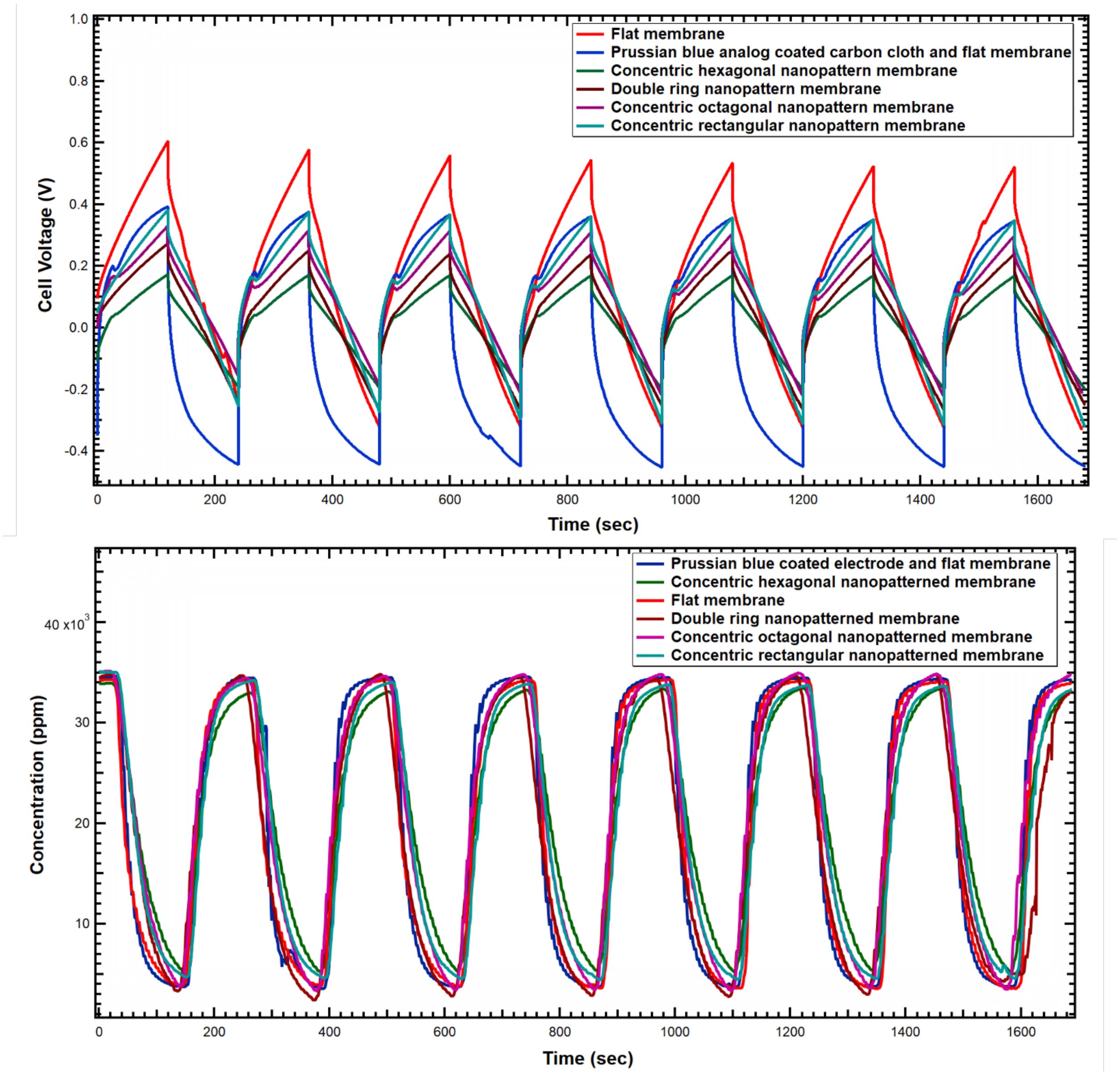

Figure S12. a) Cell voltage versus time and b) effluent concentration profiles versus time for charge-discharge cycles in MCDI at a current density of 2 mA cm^{-2} for a mixture of 30,000 ppm NaCl and 5,000 ppm $MgSO_4$ feed using flat and nanopatterned IEMs. Both the charge (deionization) and discharge (regeneration) steps were 120 seconds each. The cell geometric projected area was 25 cm^2. Seven cycles from the chronopotentiometry experiments are shown here.

Section S11. Effect of charge/discharge time on the kinetics of hexagonal nanopatterned membrane experiments

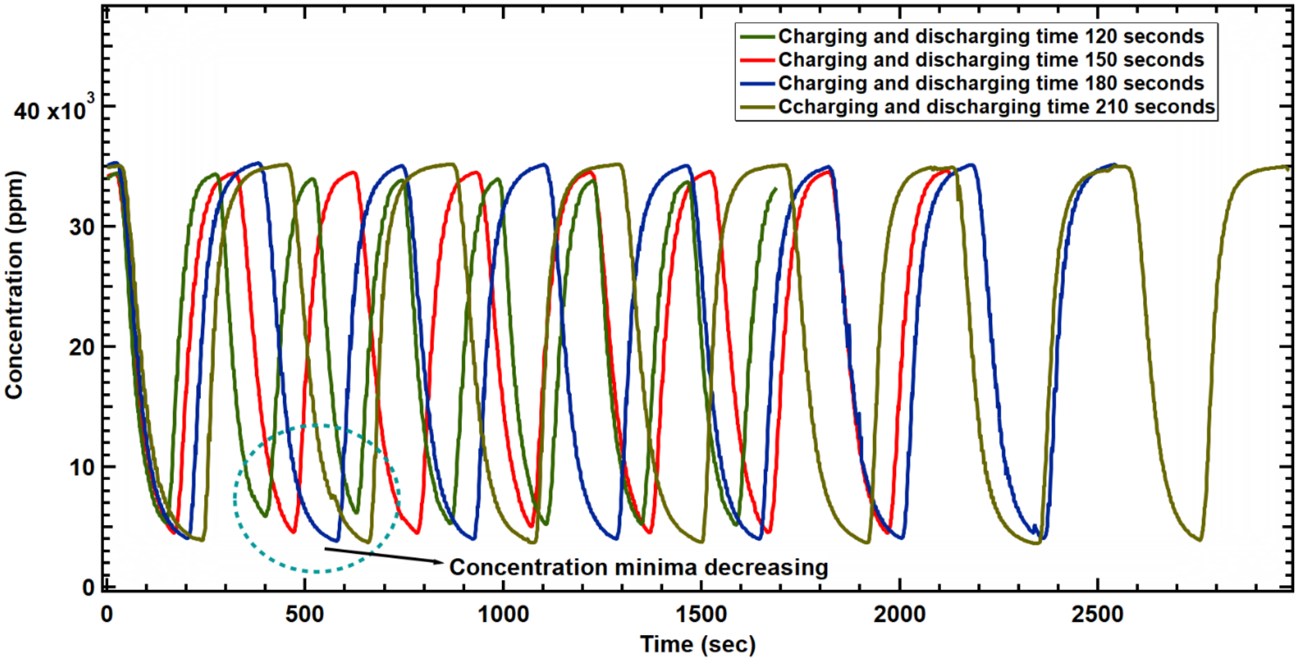

Figure S13. Effect of charge/deionization and discharge/regeneration time on the deionization of 35,000 ppm NaCl for hexagonal nanopatterned membranes at current density of 2 mA cm^{-2}. The dashed circle show that concentration minima is decreasing when charge and discharge times are increased from 120 seconds to 210 seconds.

Section S12. SI Equivalent circuit model and parameters from equivalent circuit modeling of EIS experiments

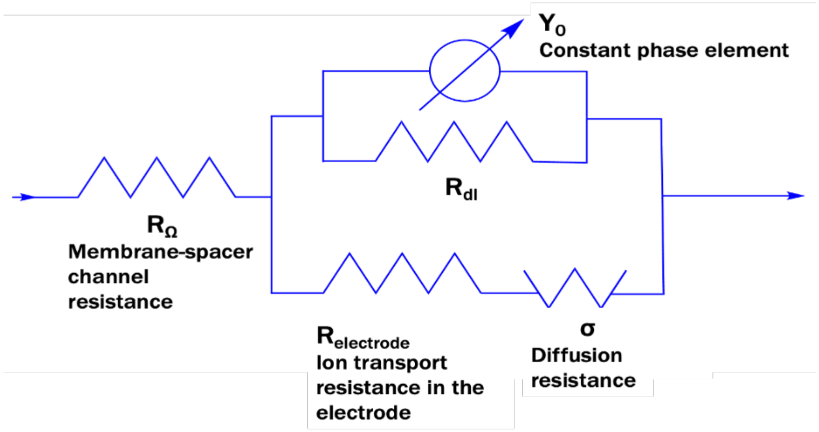

Figure S14: Equivalent circuit model used to deconvolute resistance in the MCDI system

Section S13. Electrode stability data for MCDI with hexagonal nanopatterned membranes

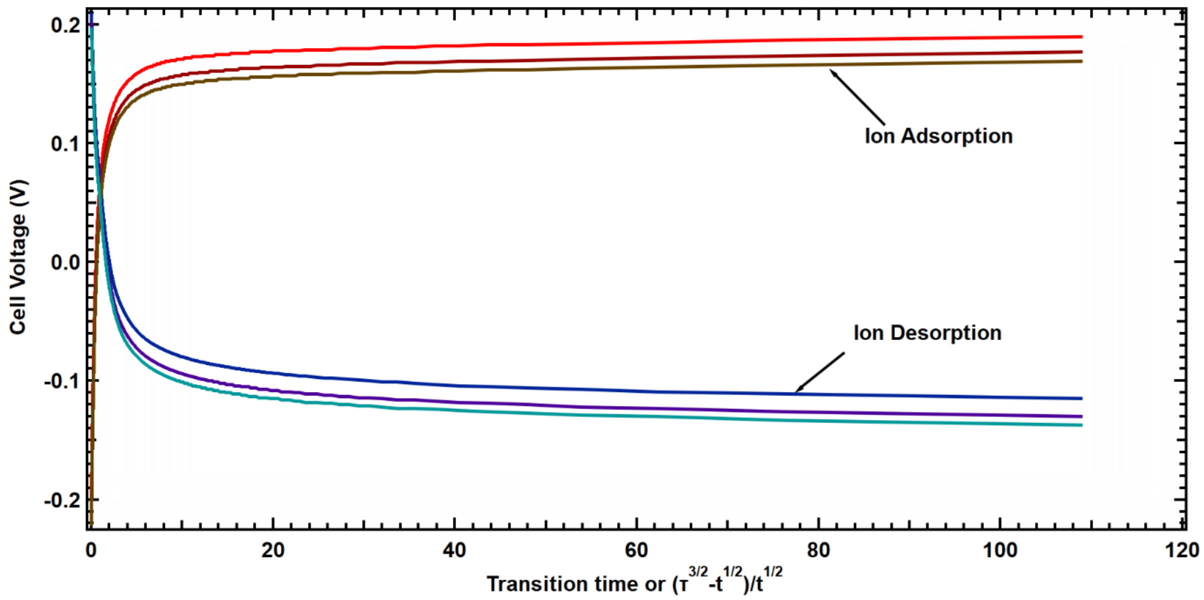

Figure S15. Electrode stability data for three cycles of adsorption and desorption process in MCDI when hexagonal nanopatterned membrane and carbon cloth electrode is used. Here, the feed was 35000 ppm NaCl and 2000 ppm NaCl was used as dilute regeneration solution.

Table S2. Membrane and spacer channel ohmic resistance values for different cell configurations

Cell configuration	R_Ω ($\Omega.cm^2$) value
Concentric hexagonal nanopatterned membrane	14.48
Double ring nanopatterned membrane	31.25
Concentric octagonal nanopatterned membrane	24.7
Concentric rectangular nanopatterned membrane	40.325
Flat membrane	57

Table S3. Salt removal efficiency (SRE), average salt adsorption rate (ASAR), and water recovery (WR) for DI water flushing of different high-sainity feeds with flat IEMs and porous carbon electrodes. at a current density of 2 mA cm^{-2}.

Feed type	SRE [%]	ASAR [mmol. m^{-2}. s^{-1}]	WR [%]
35,000 ppm NaCl	97.12	38.00	39.20
30,000 ppm NaCl and 5,000 ppm MgSO4 mixture	96.90	38.30	38.20
30,000 ppm NaCl	98.73	25.69	32.63
5,000 ppm $MgSO_4$	96.20	5.25	38.20

Table S4. Salt removal efficiency (SRE), average salt adsorption rate (ASAR), and water recovery (WR) during flushing with 0–5000 ppm NaCl for a 35,000 ppm NaCl solution using flat IEMs and porous carbon electrodes at a current density of 2 mA cm^{-2}.

Regeneration solution concentration [ppm]	SRE [%]	ASAR [mmol. m^{-2}. s^{-1}]	WR [%]
0	97.12	38.00	39.20
250	97.74	38.20	33.33
500	93.00	32.10	33.83
1,000	93.02	34.10	33.62
2,000	89.74	44.02	34.27
5,000	77.23	27.51	28.72

Table S5. Salt removal efficiency (SRE), average salt adsorption rate (ASAR), and water recovery (WR) during flushing with 1,700 ppm NaCl and 300 ppm $MgSO_4$ for a 30,000 ppm NaCl and 5,000 ppm $MgSO_4$ feed using flat IEMs and porous carbon electrodes at 2 mA cm^{-2}.

Feed type	Flushing type	SRE [%]	ASAR [mmol. m^{-2}. s^{-1}]	WR [%]
mixture of 30,000 ppm NaCl and 5,000 ppm $MgSO_4$	1,700 ppm NaCl and 300 ppm $MgSO_4$	89.11	56.36	48.42
30,000 ppm NaCl	1,700 ppm NaCl	90.59	37.33	33.34
5,000 ppm $MgSO_4$	300 ppm $MgSO_4$	98.19	5.65	33.54

Table S6. Salt removal efficiency (SRE), energy recovery (ER), energy normalized adsorbed salt (ENAS), average salt adsorption rate (ASAR), and water recovery (WR) for flat IEMs, nanopatterned IEMs and PBA-infiltrated electrodes with a feed of 35000 ppm NaCl.

**Cell configuration**	**SRE [%]**	**ASAR [mmol m^{-2} s^{-1}]**	**ER [%]**	**ENAS [mmol J^{-1}]**	**WR (%)**
Flat membrane and carbon cloth electrode	89.74	44.02	33.80	64.00	38.40
Flat membrane and Prussian blue coated electrode	88.70	39.90	43.40	115.50	36.11
Concentric rectangle nanopatterned membrane and carbon cloth electrode	89.51	45.18	31.10	146.50	37.40
Double ring nanopatterned membrane and carbon cloth electrode	89.60	42.32	47.40	317.30	35.90
Concentric octagon nanopatterned membrane and carbon cloth electrode	90.90	40.92	30.50	146.80	34.30
Concentric hexagon nanopatterned membrane and carbon cloth electrode	87.52	47.35	28.73	382.10	36.10

Table S7. Salt removal efficiency (SRE), energy recovery (ER), energy normalized adsorbed salt (ENAS), average salt adsorption rate (ASAR), and water recovery (WR) for flat IEMs, nanopatterned IEMs and PBA-infiltrated electrodes with a feed of mixture of 30000 ppm NaCl and 5000 ppm $MgSO_4$

Cell configuration	SRE [%]	ASAR [mmol. m^{-2}. s^{-1}]	ER [%]	ENAS [mmol J^{-1}]	WR (%)
Flat membrane and carbon cloth electrode	89.10	36.60	31.62	61.20	34.14
Flat membrane and Prussian blue coated electrode	89.30	39.50-	36.01	123.90	35.80
Concentric rectangle nanopatterned membrane and carbon cloth electrode	88.00	47.60	30.30	149.30	37.34
Double ring nanopatterned membrane and carbon cloth electrode	90.70	39.82	32.53	167.60	36.44
Concentric octagon nanopatterned membrane and carbon cloth electrode	89.30	42.47	31.10	141.20	35.83
Concentric hexagon nanopatterned membrane and carbon cloth electrode	86.60	44.81	25.60	423.40	36.41

Section S14. Effluent concentration with and without regeneration for 35000 ppm NaCl

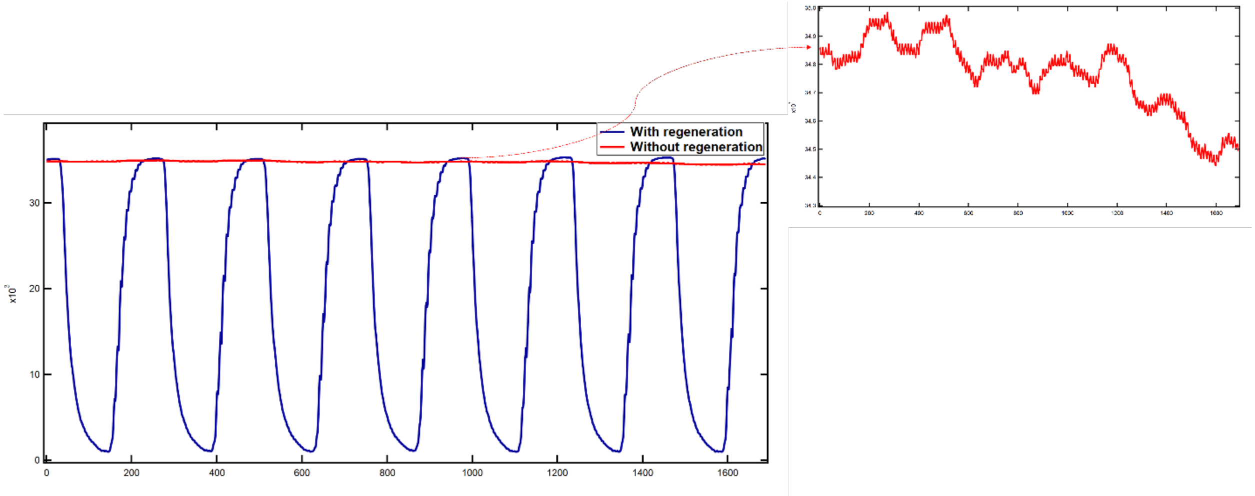

Figure S16. Effluent concentration for 35000 ppm NaCl feed with (2000 ppm NaCl) and without regeneration (35000 ppm NaCl).

Section S15. Long term cycling of carbon cloth and PBA analog coated electrodes

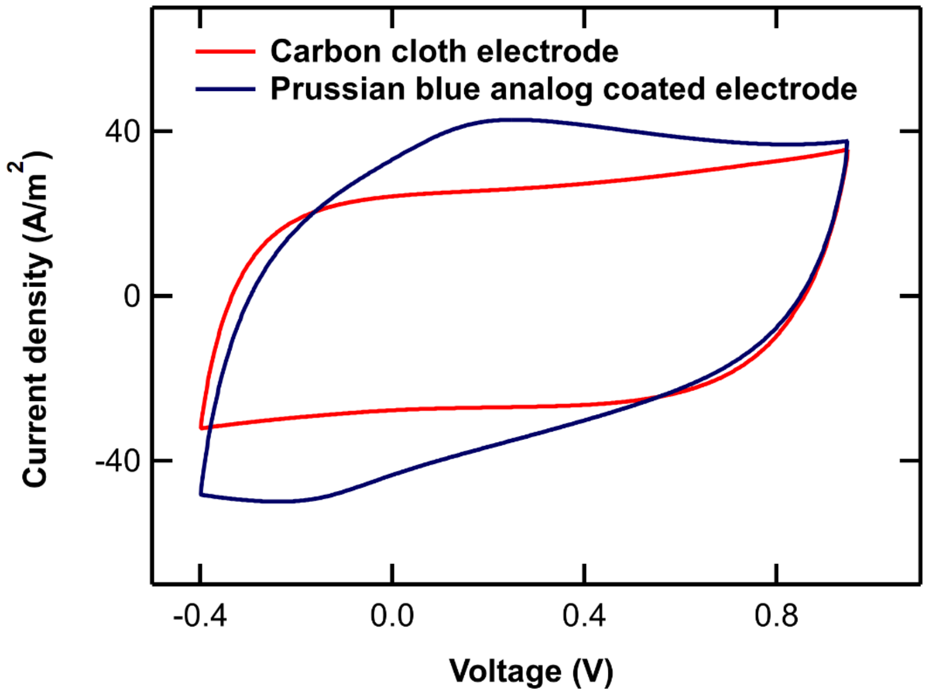

Figure S17: Cyclic voltammogram of carbon cloth electrodes and Prussian blue analog coated electrodes

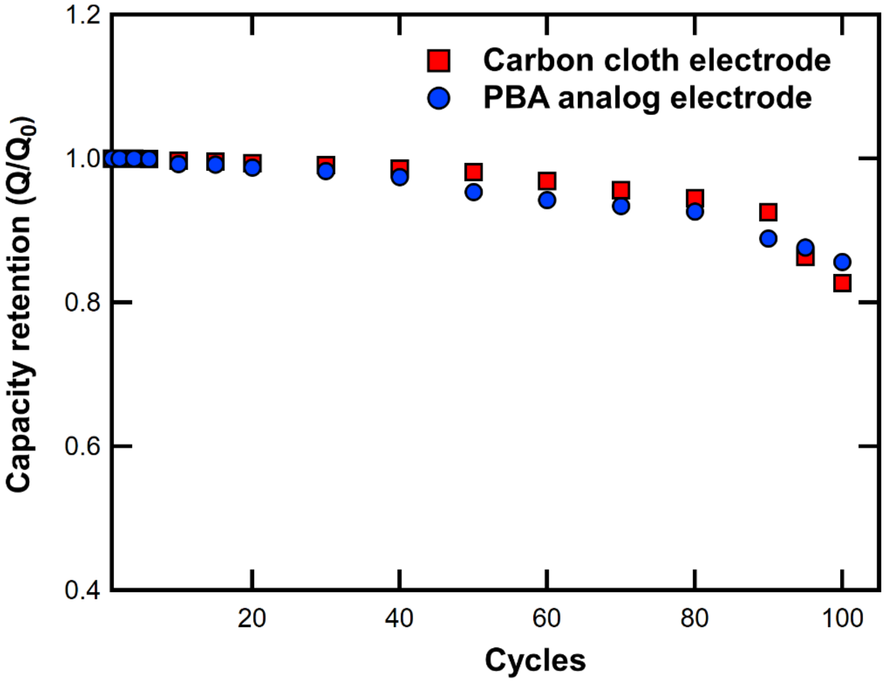

Figure S18: Long term cycling of carbon cloth electrodes used for desalination of 35000 ppm NaCl
